# Aptamers for Proteins Associated with Rheumatic Diseases: Progress, Challenges, and Prospects of Diagnostic and Therapeutic Applications

**DOI:** 10.3390/biomedicines8110527

**Published:** 2020-11-22

**Authors:** Elizaveta A. Shatunova, Maksim A. Korolev, Vitaly O. Omelchenko, Yuliya D. Kurochkina, Anna S. Davydova, Alya G. Venyaminova, Mariya A. Vorobyeva

**Affiliations:** 1 Institute of Chemical Biology and Fundamental Medicine, Siberian Division of Russian Academy of Sciences, 630090 Novosibirsk, Russia; lizashatunova@yandex.ru (E.A.S.); anna.davydova@niboch.nsc.ru (A.S.D.); ven@niboch.nsc.ru (A.G.V.); 2 Research Institute of Clinical and Experimental Lymphology, Affiliated Branch of Federal Research Center of Cytology and Genetics, Siberian Division of the Russian Academy of Sciences, 630060 Novosibirsk, Russia; kormax@bk.ru (M.A.K.); v.o.omelchenko@gmail.com (V.O.O.); juli_k@bk.ru (Y.D.K.)

**Keywords:** aptamers, rheumatic diseases, protein biomarkers, aptasensors, aptamer therapeutics

## Abstract

Nucleic acid aptamers capable of affine and specific binding to their molecular targets have now established themselves as a very promising alternative to monoclonal antibodies for diagnostic and therapeutic applications. Although the main focus in aptamers’ research and development for biomedicine is made on cardiovascular, infectious, and malignant diseases, the use of aptamers as therapeutic or diagnostic tools in the context of rheumatic diseases is no less important. In this review, we consider the main features of aptamers that make them valuable molecular tools for rheumatologists, and summarize the studies on the selection and application of aptamers for protein biomarkers associated with rheumatic diseases. We discuss the progress in the development of aptamer-based diagnostic assays and targeted therapeutics for rheumatic disorders, future prospects in the field, and issues that have yet to be addressed.

## 1. Introduction

**Figure 1:**
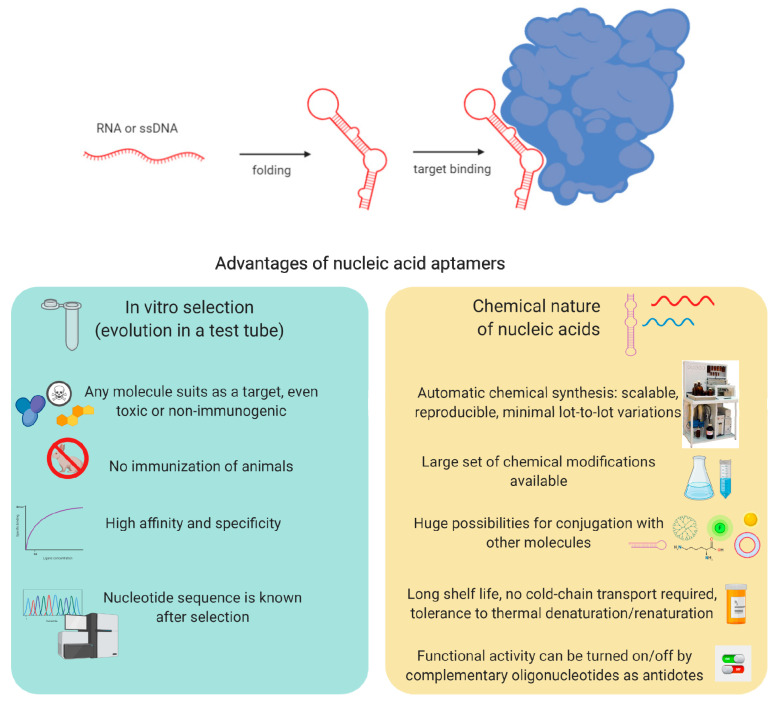
Schematic representation of aptamer’s folding and target recognition, and the main advantages of nucleic acid aptamers.

Nucleic acid aptamers are small DNA or RNA fragments capable of tight and specific binding to their molecular targets due to the formation of the spatial structure, providing a unique landscape of aptamer-target contacts (schematically represented in Figure 1). In the early 1990s, three independent research groups proposed the technology for generating aptamers, which is now widely known as SELEX—Systematic Evolution of Ligands by Exponential Enrichment [1,2,3]. Conceptually, SELEX represents an “evolution in the test tube” right on a lab bench. Aptamers are the closest analogs of monoclonal antibodies in terms of binding affinity and specificity. However, aptamers offer a number of their very own advantages (summarized in Figure 1, lower panel). Generation of nucleic acid aptamers does not require immunization of animals and allows for selection against any target molecule, even toxic or non-immunogenic. DNA/RNA aptamers are nucleic acids with established nucleotide sequences, so they can be synthesized using an automatic DNA/RNA synthesizer in the lab or by a commercial company. Chemical synthesis, in turn, provides stable properties of the aptamers (binding affinity and specificity) with minimal lot-to-lot variations and gives the widest possibilities to introduce different chemical modifications for improving binding properties, biological stability, or pharmacokinetic profiles. Moreover, aptamers can be conjugated with reporter groups, drugs, or nanoparticles. Predictable properties and secondary structure make them convenient building blocks for incorporation into different multi-component molecular constructs. The functional activity of nucleic acid aptamers can be easily turned on/off by complementary oligonucleotides as antidotes [4]. Finally, aptamers are tolerant to long-term storage and multiple thermal denaturation/renaturation cycles and do not require cold-chain transport.

Thanks to these benefits, aptamers now represent a generally acknowledged alternative to monoclonal antibodies. To date, aptamers have been selected to a huge variety of molecular targets, from small molecules to viruses and cells (see, e.g., the review [5]). Among them, protein targets are of particular interest for biomedicine. Aptamers against disease-related proteins can modulate their functional activity in an inhibitory, antagonistic, or agonistic manner, which offers a possibility to develop aptamer-based targeted therapeutics [6]. About 30 aptamer therapeutics are currently undergoing clinical trials, with one aptamer drug, Macugen (Pfizer), approved for the market [7,8].

On the other hand, aptamers recognizing biomarkers serve as biospecific elements for diagnostic systems. Relatively small molecular size, huge possibilities of chemical modification, and compatibility with various biosensing platforms have brought to life an enormous repertoire of aptamer-based detection systems, from fast and straightforward point-of-care assays to sophisticated schemes and devices [8,9,10,11]. Aptamer-based detection systems are now on the way to clinical applications, with several examples of commercial aptamer-based assays [12,13]. However, we would like to make an accent on the great potential of aptamer-based diagnostic assays and their benefits over ubiquitous antibody-based techniques, such as ELISA. In recent years, researchers have tended to report the shortcomings and limitations of diagnostic antibodies, which engender a problem of reproducibility of the results. The affinity and specificity of antibodies vary between different vendors, and even the concrete antibody from a particular supplier can suffer from lot-to-lot variations [14,15]. Outside of clinical trials, antibodies are rarely characterized and validated to a degree that provides a high reliability. A problem of insufficient reproducibility is particularly acute in the cases of long-term studies relying on certain antibodies that can become unavailable to the manufacturer after some years [16]. A. Bradbury, with co-authors (more than a hundred co-signers) in their publication in Nature, suggested standardizing the antibodies [15]. This means that monoclonal antibodies should be produced recombinantly, and their sequence information should be used as a universal reference system for the researchers choosing the binding agent. Otherwise, aptamers readily meet the abovementioned requirements. Their nucleotide sequences are stored as digital information. They are always available for chemical synthesis, which is not affiliated with one particular manufacturer, so nearly any chosen aptamer can be obtained any time on request. Moreover, chemical synthesis guarantees minimal lot-to-lot variations, providing high reproducibility for aptamer-based studies. Therefore, aptamers seem to be very promising tools to solve the “reproducibility crisis.”

**Figure 2:**
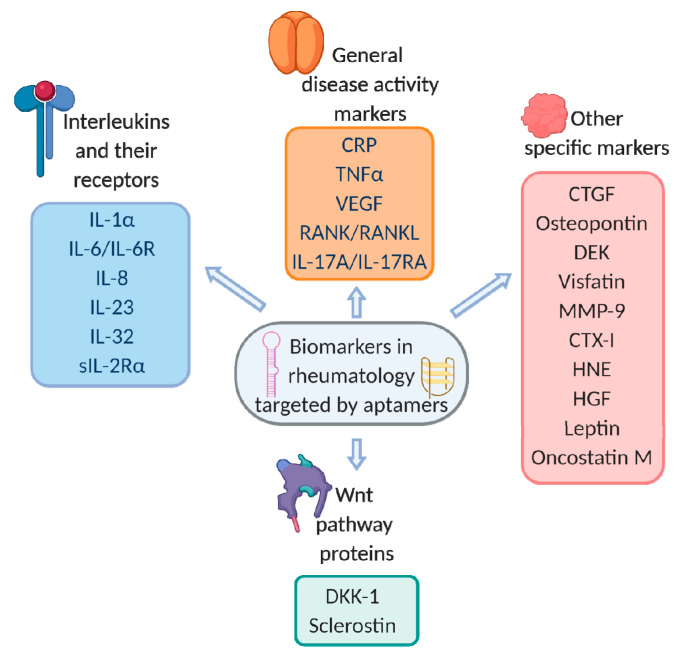
Protein biomarkers in rheumatology currently targeted by aptamers.

Until now, studies on aptamers’ application for therapy and diagnostics have tended to focus mostly on cardiovascular, malignant, and infectious diseases (see, e.g., the reviews [8,17,18,19,20]). However, other medicinal disciplines would benefit as well from implementing aptamer-based technologies. In particular, we would like to consider this problem in the context of rheumatic disorders. These pathologies show a high prevalence in the general population (only rheumatoid arthritis affects at least 1% of the world’s population) and greatly impact health-related life quality. Modern therapeutic approaches for treating rheumatic disorders imply the use of antibody-based drugs targeted to proteins related to general inflammation or more specific signalling pathways [21]. The early diagnostics and monitoring of treatment efficiency requires also the quantitative evaluation of protein biomarkers. Such nonspecific markers as CRP or TNFα serve as precursors of complications and are decreased during remission or low activity of any rheumatic disease [21,22,23]. At the same time, evaluation of the specific markers such as IL-17 in spondylitis can be used both for early diagnostic and for verification of low disease activity achievements [24]. More than that, the chronic nature of rheumatic diseases demands long-term studies with repeated monitoring of key biomarkers [25], which, in turn, requires reliable diagnostic assays. In the present review, we systemize the data concerning the aptamers specific to proteins associated with rheumatic diseases (summarized in Figure 2), the current status of their diagnostic and therapeutic applications, and future prospects in the field.

## 2. Selection and Chemical Modifications of Nucleic Acid Aptamers

**Figure 3:**
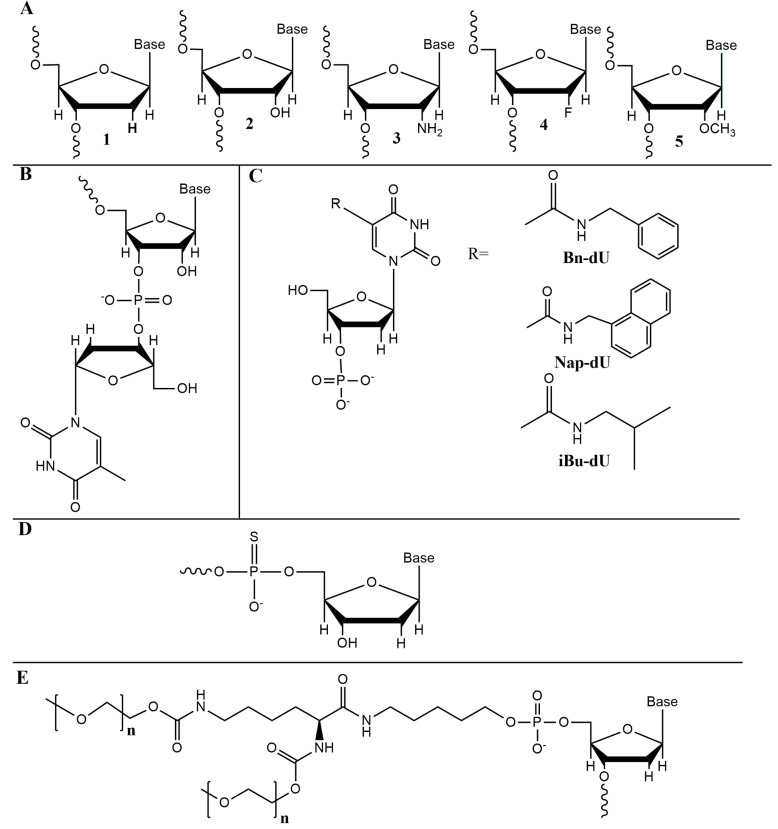
Chemical modifications employed for the aptamers under review. (**A**) Natural deoxyribo (1) and ribonucleosides (2), 2′-amino-2′-deoxyribonucleoside (3), 2′-fluoro-2′-deoxyribonucleotide (4), and 2′-O-methylribonucleotide (5). (**B**) An “inverted” 3′-thymidine attached by 3′-3′-phosphodiester linkage. (**C**) Examples of hydrophobic modifications of heterocyclic bases used in SOMAmers [29]. (**D**) Phosphorothioate analogs of oligodeoxyribonucleotides. (**E**) 5′-PEG-modified (PEGylated) aptamer.

A general SELEX scheme (Figure 3) includes the generation of a combinatorial DNA or RNA library (10^11^–10^15^ sequences), its incubation with the target, partitioning of aptamer-target complexes from unbound nucleic acids, isolation of bound aptamers, and their amplification. The resulting enriched library goes to the next round of selection. The final enriched library after approximately 7–12 rounds of selection is sequenced to identify the individual molecules. Nowadays, high-throughput sequencing methods are most popular for this purpose. After bioinformatic analysis of sequencing data, the most promising candidate aptamers are synthesized and tested for their affinity and specificity to reveal the best binder. A large variety of SELEX methods developed to the moment [26,27,28] allows for choosing the most suitable protocol for any research task. Specifically, the type of the library (DNA, RNA, or their SELEX-compatible modified analogs), its manner of randomization, length, and secondary structure can also be altered depending on the particular target and the intended use of the aptamer [29,30].

As a rule, an additional post-selective design of the aptamer includes removing nucleotides, which are not necessary for target binding. Aptamer truncation reduces the probability of non-specific binding and lowers the cost of manufacturing. A no less important part of the post-selective design is the introduction of chemical modifications which improve the nuclease resistance of the aptamer, such as replacement of natural ribo/deoxyribonucleotides by their sugar (Figure 3A) and/or phosphate-modified analogs (Figure 3D), and the addition of the “inverted” 3′-terminal thymidine residue attached via unnatural 3′-3′ phosphodiester linkage (Figure 3B). Some examples of chemical modifications of the aptamers described in this review are given in Figure 3. A detailed discussion of aptamers’ chemical modifications can be found in recent comprehensive reviews [6,29,31,32].

While aptamers employed in diagnostic assays should meet only the requirement of nuclease resistance, aptamers derived for therapeutic applications face the additional problem of systemic clearance through renal filtration because of their relatively low molecular weight (~20 kDa). The attachment of 40-kDa hydrophilic polymer polyethylene glycol (PEG) to the 3′- or 5′-end (so-called PEGylation) (Figure 3D) is the most widely used strategy to increase aptamer’s circulation time and enhance its pharmacodynamic properties [33]. Such polymers have been generally considered biologically inert; nevertheless, severe allergic reactions to PEGylated therapeutic aptamers were reported (see the reviews [6,32]). This problem originates from anti-PEG antibodies, which could be generated with repeated administration, and can potentially occur not only with aptamers, but with any PEGylated compound [33]. The issues of anti-PEG immunity and alternatives to PEG were thoroughly reviewed by Zhang et al. [34]

## 3. Aptasensors—Aptamer-Based Bioanalytical Systems

Aptamer-based biosensors, also referred to as aptasensors, are analytical devices consisting of a biorecognition element (aptamer) and a transducer that provides a quantitative or semi-quantitative analytical signal upon analyte binding. As we mentioned above, a huge variety of aptasensing platforms have been developed to the moment for the detection of analytes related to food safety [35,36], environmental studies [37,38,39], and clinical diagnostics [19,40,41]. Most of them rely on optical (colorimetrical, fluorescent, luminescent, or surface plasmon resonance (SPR)) [9,10,39] and electrochemical (current, conductance, potential, and impedance) [42,43] types of the analytical signal.

**Figure 4:**
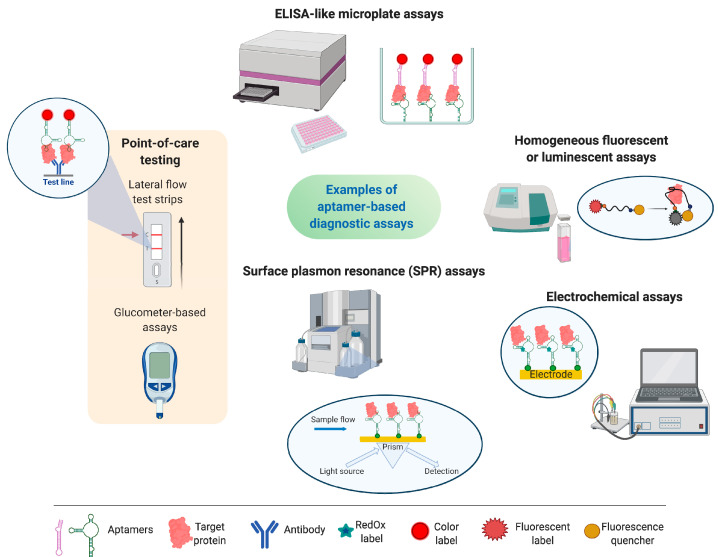
Examples of aptamer-based diagnostic assays and corresponding devices.

It should be noted that aptasensors intended to be used in clinical diagnostic assays should meet a set of criteria for their successful application. High specificity and sensitivity of detection are undoubtedly significant, especially for rheumatological biomarkers, which are commonly presented in biological fluids in low (pg/mL or ng/mL) concentrations. However, just as important are minimal sample pre-processing, fast and simple detection protocol, compatibility with standard equipment of the clinical lab, good reliability, and reproducibility of the results. A very interesting field in the aptasensors’ engineering is represented by point-of-care (PoC) aptasensors which allow for rapid on-site testing outside the laboratory, such as lateral flow test strips [44] or devices based on personal glucometer, portable pH meter, or even a smartphones (see, e.g., the recent review [45]). The examples of aptasensors for detecting rheumatological biomarkers described in this review are given in Figure 4.

## 4. Aptamers for Protein Biomarkers of Rheumatic Disorders

### 4.1. General Disease Activity Markers

#### 4.1.1. C-Reactive Protein (CRP)

C-reactive protein (CRP) is an established marker for disease activity measurement of inflammatory arthritis both in clinical practice [21,23,46,47] and in research studies [48,49]. Elevated CRP levels can be revealed before the clinical manifestation of inflammatory arthropathy [50]. The evaluation of the activity of systemic vasculitis are also based on the measurement of CRP levels [51,52]. An increased CRP level may be a predictive marker of systemic scleroderma progression for both skin lesions and pulmonary fibrosis [53,54]. CRP evaluation is used to exclude the development of infectious complications in systemic lupus erythematosus (SLE) [55,56]. High CRP levels are directly connected with the severity and speed of radiographic progression in males with ankylosing spondylitis (AS) [22] and in rheumatoid arthritis (RA) patients without regard to gender [57]. Increased CRP, along with the evaluation of alkaline phosphatase levels, allow predicting osteoporosis (OP) progression and spinal fracture risks in RA patients [58]. Increased CRP level is also an established predictive risk marker of cardiovascular complications and thrombosis [59,60,61,62,63]. CRP level indicates the efficacy of background anti-inflammatory therapeutics and biologics, successfully applied either in clinical practice [64,65,66] or undergoing clinical trials [67,68,69,70]. It is well-known that patients with higher CRP levels show a better therapeutic response [71,72].

##### Aptamer-Based CRP Detection Assays

So far, a wide range of aptamer-based assays have been developed for quantitative measurement of CRP levels. Bini et al. employed a 44-mer RNA aptamer to develop an SPR-based CRP detection system [73] (here and after, nucleotide sequences of aptamers and corresponding K_D_ values are given in Table 1). The aptamer was immobilized onto the gold chip surface for SPR detection of the CRP with the limit of detection of 500 ng/mL and linear range up to 1000 ng/mL (here and after, see Table 2 for the summary of aptasensors and their characteristics). The sensor showed the specificity to CRP, compared to HSA and IgG taken as controls. Model serum samples were represented by a mixture of CRP (0.01 ppm), IgG (240 ppm), and HSA (500 ppm). The samples were treated by protein G magnetic beads and diluted (1:2) to obtain the protein concentrations corresponding to those in 1:100 diluted human serum.

The same RNA aptamer found numerous applications in further works on aptasensor development. Qureshi et al. [109] reported a label-free electrochemical aptasensor for CRP detection. Gold electrodes were functionalized by 5′-thiolated 44-nt RNA aptamer, and non-Faradaic impedance spectroscopy was applied for monitoring aptamer-CRP binding. The sensor detected CRP in the range of 100–500 pg/mL and demonstrated CRP binding specificity compared to BSA.

The aptamer, immobilized on the gold electrode through a 5′-thiol group, formed a recognition layer in the electrochemical aptasensor based on square-wave voltammetry with a methylene blue as a redox indicator [110]. The sensor gave a linear response from 25 to 250 pg/mL and a good specificity to CRP compared with BSA and IgE (as model interfering proteins). The authors also demonstrated a principal possibility of CRP detection in a 10% serum sample spiked with the protein. Of note, the performance in serum decreased significantly because of the adsorption of serum components on the electrode surface.

**Figure 5:**
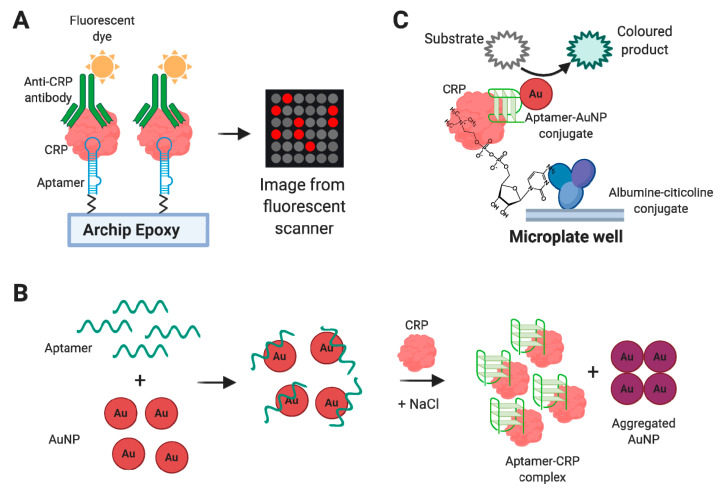
Examples of aptasensors for C-reactive protein: aptamer-based chip for fluorescent sandwich immunoassay (**A**) [111], colorimetric assay based on AuNPs aggregation (**B**) [112], and ELISA-like system employing citicoline for CRP capture and peroxidase-mimicking AuNPs [113] (**C**).

Pultar et al. [111] engineered an RNA aptamer-based biochip for a fluorescent sandwich immunoassay. The aptamer was immobilized on an epoxy-modified microchip, and bound CRP was detected by using fluorescently labeled anti-CRP antibodies on a Genepix^TM^ 4000B scanner (Figure 5A). The limit of detection in a buffer was 1.6 ng/mL. Aptamer/antibody sandwich chips demonstrated the working range in spiked serum from 10 to 100 µg/mL. This range allows determining both normal and elevated CRP concentrations with only one sample dilution (if necessary). Of note, the aptamer-based system provided much better performance than the analogous antibody/antibody chip, which was unable to measure concentrations >1 µg/mL.

The sandwich system for electrochemical detection developed in [114] contained a 44-nt 2′-F-Py RNA aptamer immobilized on magnetic beads through biotin-streptavidin interactions and anti-CRP antibody conjugated with alkaline phosphatase. The authors used a uniform 2′-fluoro modification for RNA aptamer to enhance its serum stability. After the sandwich assembly and transferring of the beads to the disposable screen-printed electrode, the enzymatic substrate was added, and the product was determined by differential pulse voltammetry. In the model solution, the system provided a specific signal (compared to human IgG control) in the detection range of 0.1–50 µg/mL. The electrochemical aptamer-based assay also demonstrated an ability to quantitatively detect CRP in 1:10 diluted serum spiked by 0–1000 µg/mL CRP. Testing two clinical serum samples from healthy individuals gave reasonably low CRP levels, making this sensor promising for a quantitative CRP measurement in clinical samples.

Alternatively, Wang et al. [115] employed the functionalized silica microspheres to make an RNA aptamer-based electrochemical sandwich aptasensor with square wave voltammetry detection. The aptamer bearing a 5′-SH group was assembled on the Au nanoparticles-modified electrode surface. The immunoprobe for CRP detection consisted of the silica microspheres decorated by anti-CRP antibodies and signal Zn^2+^ ions. Under optimal conditions, the working range of the system was 0.005–125 ng/mL. The sensor showed good specificity to CRP compared to the prostate-specific antigen, α-fetoprotein, and carcinoembryonic antigen. The analysis of clinical samples (1:500 diluted serums) gave the same CRP levels as a reference immunofluorescence assay, thus demonstrating the potential applicability of this aptasensor.

Bernard et al. [116] developed a sandwich aptamer/antibody system for fluorescent CRP detection. In this case, RNA aptamer-modified magnetic beads were placed into microplate wells, and the analyte was detected on the Luminex platform by a biotinylated anti-CRP antibody coupled to a fluorescent phycoerythrin/streptavidin conjugate. The assay provided quantitative CRP detection in the range of 0.4–10 µg/mL for spiked serum samples diluted 1:100, demonstrating that serum levels from approximately 40 to 1000 µg/mL could be measurable in clinical samples. However, this assay was unable to quantify low CRP levels (<10 µg/mL).

An alternative CRP-binding 104 nt RNA aptamer was reported by Orito et al. [74] in 2012. Despite its high affinity (K_D_ = 2.3 nM), this aptamer still has not found any analytical applications, most probably because its nucleotide sequence is too long for the design of aptasensors, and needs rational truncation for further use.

C. Eid [117] et al. reported a SOMAmer-based system for fluorescent CRP detection by the on-chip electrophoretic assay. They used an isotachophoresis in the presence of spacer ions to both react and separate the SOMAmer-CRP complex from a free SOMAmer. The assay protocol took only 20 min, and the limit of detection in buffer was 50 ng/mL with a 2.5-decade dynamic range. It is worth noticing that the visualization of the aptamer-target complex required a fluorescence microscope, so the whole system can hardly be applied for standard lab diagnostics. The sensor also allowed CRP detection in spiked serum samples (diluted 1:20), although, in this case, the LOD increased up to 625 ng/mL. The authors mentioned that serum ions (such as phosphate, sulfate, bicarbonate, and uric acid) could decrease the assay’s performance, and the presence of abundant serum proteins, particularly albumin, can also increase the background signal due to non-specific binding.

A 71 nt DNA aptamer Clone 1 with high CRP-binding affinity (K_D_ = 3.5 nM) was generated by Huang et al. [75] A chemiluminescent sandwich assay with aptamer-functionalized magnetic beads, acridinium ester-labeled anti-CRP antibodies, and HNO_3_/H_2_O_2_ treatment provided a linear range of detection from 0.0125 to 10 mg/mL in a buffer. The authors also integrated a Clone 1 aptamer into a field effect transistor (FET) microfluidic device for CRP detection [118]. The aptamer was immobilized on the Au gate of the FET device through a 5′-thiol group. This assay provided CRP detection in a buffer with concentrations ranging from 0.625 to 10 µg/mL. A dual aptamer sandwich assay was also applicable for this system but gave a lower analytical performance.

Wu et al. [76] obtained a 40 nt G-rich DNA aptamer 6th-62-40 for CRP (K_D_ = 16.1 nM) and employed it in the SPR-based detection system. The gold SPR chip was functionalized by a 3′-thiol modified aptamer, then after CRP binding, anti-CRP coated gold nanoparticles were added to enhance the signal. The sensor demonstrated good specificity for CRP compared to other blood proteins (HSA, IgG, hemoglobin, and myoglobin) and provided an excellent detection range from 0.25 ng/mL to 2.5 µg/mL in CRP-spiked 1:100 diluted serum samples.

An ability of 6th-62-40 aptamer to form a DNA quadruplex served for a rapid fluorescent assay based on the enhanced fluorescence of thioflavin T (ThT) dye in the complex with quadruplex motifs [119]. CRP binding disrupts the aptamer/ThT complex and thus decreases the fluorescence. The method showed good specificity for CRP (compared with BSA, IgG, and myoglobin) and the working range of 12.5 ng/mL–5 µg/mL in a buffer solution.

The fast and straightforward colorimetric assay developed in [112] implies 6th-62-40 DNA aptamer and citrate-capped gold nanoparticles (AuNP) (Figure 5B). In the absence of the CRP, the aptamer adsorbs on the AuNP surface and prevents the aggregation of nanoparticles. After CRP addition, the aptamer is released from the particles and interacts preferentially with the protein, causing the aggregation of AuNPs and a subsequent red–purple color change for colorimetric detection. The assay provided a linear sensing range of 0.9–20.1 µg/mL in a buffer. It should be noted that AuNP-based assays of this type are very sensitive to the presence of serum albumin, which can cause the aggregation of AuNPs. The authors demonstrated principal applicability of the assay in model solutions with low albumin concentration (0–6.6 µg/mL) and in 1:100 diluted CRP-spiked urine samples. It remains questionable whether this detection is suitable for serum samples, which contain very high albumin concentrations (approximately 35 mg/mL).

Xie et al. [113] recently proposed a very promising sandwich ELISA-like assay for CRP detection (Figure 5C). The method employs the ability of CRP to bind specifically with choline phosphate. Citicoline (cytidine 5′-diphosphocholine) coupled with BSA became a plate-coating CRP-specific molecule. The second component of the sandwich was represented by AuNPs with a 6th-62-40 DNA aptamer immobilized through a 3′-thiol group. AuNPs exhibited peroxidase activity and oxidized chromogenic substrate tetramethylbenzidine (TMB), giving a blue color for the quantitative measurement. The assay demonstrated the working range of 0.1 to 200 ng/mL with an excellent specificity in the presence of different potentially interfering blood components, including BSA, myoglobin, troponin 1, amino acids, and glucose. Due to the good stability between batches, the same calibration curve could be used to calculate the sample content in different batches, which is the advantage of the assay over the classical ELISA. Notably, the method was applied to analyze real blood samples (diluted 1:100) and showed perfect agreement with the results obtained by commercial kits.

Yang et al. [77] used the GO-SELEX technique to generate the CRP-binding 79 nt DNA aptamer CRP-80-17. At the moment, there was only one attempt to use this aptamer for bioanalytical purposes. The aptamer adsorbed on the optic fiber coated by indium tin oxide film was employed for homogeneous CRP detection by a high sensitive refractometer [120]. The method demonstrated excellent sensitivity (LOD of 0.0625 µg/mL) in a buffer solution, but was not tested in the model or real biological samples.

#### 4.1.2. Tumor Necrosis Factor Alpha (TNFα)

TNFα is a pro-inflammatory cytokine initially discovered in 1975 as an endotoxin-induced factor. TNFα regulates plenty of biological processes, such as proliferation, differentiation, and death of different cells, inflammatory responses, and innate and acquired immunity [143]. It also participates in the structure formation of different organs and tissues, including secondary lymphoid organs. Uncontrollable increased TNFα production can induce pathological processes, e.g., septic shock during infectious or chronic inflammatory diseases [144]. TNFα is released as a response to different stimuli by immune cells, including monocytes, macrophages, dendritic cells, T- and B-lymphocytes, mast cells, as well as stromal cells, neural system cells, and skin and endothelial cells. Activated cells produce TNFα as a transmembrane protein, which is later separated from the cell surface by metalloproteases, mainly TNFα converting enzyme (TACE), and is then secreted as a soluble protein [145]. Both TNFα forms are biologically active as homotrimers and induce intracellular signaling through binding cell surface TNF-receptors 1 and 2 (TNFR1/p55 and TNFR2/p75). TNFR1 is continuously expressed by most of the organism cells, while the expression of TNFR2 is activation-dependent and is observed mostly in immune and endothelial cells [145]. The study on transgenic mice revealed that increased TNFα production leads to spontaneous development of chronic inflammatory processes in different organs and tissues similar to RA, AS, inflammatory bowel disorders, and multiple sclerosis [146,147,148]. Notably, the duration and intensity of TNFα expression in concrete cells and tissues determined certain pathology development. TNFα level correlates directly with the disease activity [149]. TNFα increases the number of osteoclast precursors in mice bloodstream and increases RANKL expression on stromal cells, thus providing the progression of erosion and osteoporosis by affecting osteoclasts [150]. The level of this cytokine is not routinely evaluated, and, as it influences plenty of cytokines, TNFα does not represent a highly specific marker for certain rheumatic diseases and could not be used for differential diagnosis. Meanwhile, the blockade of TNFα is actively used in the treatment of diseases. So far, TNFα-blocking of several therapeutics are developed to treat RA, AS, chronic bowel diseases, some central neural system, and skin diseases. Therefore, TNFα represents a widespread target for the development of therapeutics and detection systems, and its detection in serum is important for assessing the effectiveness of treatment and predicting the further course of disease.

##### Aptamer-Based TNFα Inhibitors

X. Yan et al. [80] generated, truncated, and characterized the 28 nt RNA aptamer T3.11.7 specific to TNFα and subjected it to post-selective modification by replacing all purine ribonucleotides with their 2′-NH_2_ analogs. The aptamer inhibited TNFα-dependent cytotoxicity in mouse fibroblast cell line L929 in a dose-dependent fashion, suggesting the aptamer’s utility for therapeutic applications. 

Orava et al. [79] selected DNA aptamers against TNFα, aiming to create novel inhibitors that could become an alternative for monoclonal antibodies in anti-TNFα therapy. The 25-mer aptamer VR11 bound specifically to TNFα with K_D_ = 7 nM and inhibited the interaction of TNFα with the NF-κB receptor on the HEK293T cell line. Experiments with mice fibroblasts cell line L929 proved the ability of VR11 to inhibit TNFα-mediated cytotoxicity. The authors also demonstrated on RAW264.7 macrophages that, in the presence of IFNγ and TNFα, the aptamer partially inhibits TNFα-mediated NO release and, respectively, inflammation processes. As such, VR11 DNA aptamers represent a novel non-immunogenic alternative for protein-based inhibitors of TNFα for therapy.

Very recently, two research groups reported new aptamers binding TNFα. Lai et al. [78] developed a 41 nt DNA-aptamer for specific inhibition of TNFα for inflammatory processes therapy. The resulting aptTNF-α possessed a nanomolar target binding affinity (K_D_ = 8 nM). The authors designed the dimeric PEGylated aptamer aptTNF-α-PEG for in vivo studies. Acute lung injury (ALI) and acute liver failure (ALF) mice models showed the decrease in the severity of ALI and ALF-associated symptoms and inhibition of the expression of pro-inflammatory cytokines and chemokines after single intravenous injections of the aptamer. So, aptTNF-α has the potential for therapeutic application and adds to a new category of TNF-α blocking agents.

Mashayekhi et al. [81] selected a series of anti-TNF-α DNA aptamers. The aptamers KM1, KM4, KM6, and KM8 demonstrated the highest inhibition efficiency of cytotoxic TNFα effects on mice fibroblasts. Examination of aptamer binding epitopes of TNFα revealed that KM1, KM6, and KM8 recognize the same epitope of TNFα, and KM4 binds to another epitope. Then, truncated versions of KM1 and KM4 were joined by the (dT)_10_ linker to make a new 49 nt dimeric aptamer. Its target binding affinity was higher than that of separate aptamers. In a cell cytotoxicity assay, the dimeric aptamer showed the TNFα-neutralizing effect comparable to that of Etanercept and surpassing the effect of the VR11 aptamer. This aptamer, therefore, represents a potential therapeutic and/or diagnostic agent for hTNF-α-related disorders.

##### Aptamer-Based TNFα Detection Assays

The electrochemical detection system developed on the basis of the T3.11.7 aptamer [125] comprised the phosphorothioate analog of T3.11.7 without 2′-NH_2_ modifications. The aptamer, 3′-modified with methylene blue, was immobilized on gold electrodes via the 5′-C6-disulfide linker. Signal tracking by square wave voltammetry gave the limit of detection of 10 ng/mL, with the linear range extending to 100 ng/mL both in a buffer solution and in whole TNFα-spiked blood. The sensor’s specificity was verified using the control mix of cytokines (IL-2, IL-12, IL-17, and IFN-γ). The authors also demonstrated the reusability of the detection system. The same sensor scheme was then used to fabricate the electrochemical aptasensor for the simultaneous detection of TNFα and IFNγ upon their release from cells [151]. The aptasensor successfully monitored TNFα and IFNγ release from T-cell and U937 monocytes.

Aptamer VR11 was also employed for the development of aptasensing systems. Ghalehno et al. [121] reported the electrochemical voltametric aptasensor with aptamer-modified AuNP particles, were immobilized on graphite screen-printed electrode surface. The aptasensor showed a specific signal (compared with HSA and IgG) and the linear range of 10 pg/mL–40 µg/mL in a buffer solution. This assay was also tested to analyze diluted human serum samples and compared with independent ELISA results with similar sample preparation. The results of aptasensor and ELISA analysis were close, therefore proving possible applicability of the developed aptasensor in clinical practice.

**Figure 6:**
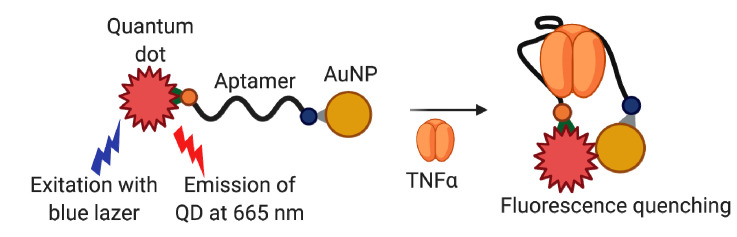
FRET-based optical aptasensor for TNFα based on the VR11 aptamer [122].

S. Ghosh et al. [122] proposed a FRET-based optical aptasensor with the use of quantum dots (QD). The aptamer VR11 conjugated to QD (fluorophore) by 5′- terminus and to AuNP (quencher) by 3′-terminus became a molecular beacon (Figure 6). After target binding, the aptamer adopts the secondary structure where fluorophore and quencher locate in close proximity, which, in turn, decreases the photoluminescent signal. The sensor showed sufficient specificity as compared with HSA, CRP, and transferrin. The working range of this system was 1.7–400 ng/mL in a buffer solution. The aptasensor demonstrated principal applicability for the analysis of diluted human sera samples spiked with TNFα. Interestingly, high cross-reactivity was observed for the aptasensor in the presence of thrombin. The authors attributed this phenomenon to the VR11 ability to form the G-quadruplex structure, which makes VR11 similar to thrombin-binding aptamer TBA. It is worth noticing, however, that the binding affinity of TBA is determined not solely by its quadruplex structure but also by the presence of specific di- and trinucleotide loops (see, e.g., [152,153]), and the overall spatial structures of these two aptamers are quite different.

Hao et al. [123] used VR11 for developing the electrochemical graphene-based field-effect transistor (GFET) aptasensor on a flexible, SiO_2_-coated substrate. The idea was to detect cytokine biomarkers (by an example of TNFα) sampled reliably from human bodily fluids (e.g., sweat) in wearable sensing applications. The aptamer was covalently immobilized on the graphene surface functionalized by 1-pyrenebutanoic acid succinimidyl ester. The binding of TNFα caused a voltage change, which allowed quantifying the biomarker concentration. Total assay time was only 5 min, and a limit of detection in a buffer solution was 0.45 ng/mL, with a good specificity compared to IFNγ and IL-2. The applicability of this sensor for biomarker detection in real sweat samples have not yet been tested.

Mayer et al. [124] proposed the aptamer-based electrochemical aptasensor which relies on the use of a redox label. The aptamer VR11, with the methylene blue attached to the heterocyclic base of T25 residue, was immobilized on a gold electrode. Voltammetric detection provided a working range of 1.75 ng/Ml–8.75 µg/mL in a buffer. The authors also showed the principal applicability of the aptasensor for the analysis of clinical samples by examples of 1:2 diluted samples of saliva and urine.

#### 4.1.3. Vascular Endothelial Growth Factor (VEGF)

Vascular endothelial growth factor (VEGF) is a biomarker for many diseases including connective tissue metabolism disorders. VEGF appears in synovial tissues in RA and participates in angiogenesis, which provides an invasion of pannus and destruction of the nearby bone tissue. VEGF’s concentration in the blood significantly increases in patients with rheumatoid arthritis and correlates with the disease activity [154]. An increase of VEGF expression along with other proangiogenic factors is noticed for psoriatic arthritis. It was more prominent compared to RA and associated with different morphology of blood vessels [155], so this marker can be useful to differentiate these diseases.

Since VEGF is involved in the pathogenesis of different diseases, it attracted particular attention as a SELEX target for further use in biomedicine. A very first FDA-approved aptamer drug against age maculodistrophy, Macugen (Pegaptanib), is based on the anti-VEGF modified RNA aptamer [156].

##### Aptamer-Based VEGF Detection Assays

The variety of aptasensors for VEGF detection is extraordinarily huge, so we only mention here the main types of them, such as luminescent, fluorescent, colorimetric, SPR- and SERS-based, and electrochemical aptasensors. Most of them provide at least pg/mL sensitivity; a more detailed discussion of VEGF aptasensors can be found in the recent comprehensive review by Dehghani et al. [157] Below are some recent examples of robust and simple colorimetric aptasensors for VEGF, which seem to be especially suitable for point-of-care diagnostics.

J. Dong et al. [126] used a 24 nt 5′-biotinylated DNA aptamer VEGF Apt1 instead of antibodies for the detection of VEGF165 in human serum. In the presence of analyte immobilized in microplate wells, VEGF165 competitively binds with aptamers and induce a decrease of the colorimetric signal generated by streptavidin-horseradish peroxidase and TMB/H_2_O_2_ system. The aptasensor provided sensitive detection of VEGF165 in a linear range 100–1 × 10^5^ pg/mL with good specificity compared to IgG, CEA, DNMT1, and BSA. The authors also tested the aptasensor’s performance in six clinical serum samples and demonstrated a reasonable correlation between their method and commercial chemiluminescence enzyme immunoassay kit. Of note, the detection protocol for the developed aptasensor did not require any pre-processing of serum samples.

S. Shan et al. [82] proposed a sandwich chemiluminescent assay based on a pair of 5′-biotinylated DNA aptamers VEGF Apt1 (24 nt) and VEGF Apt2 (26 nt) that bind different domains of VEGF165. The first aptamer was immobilized on magnetic beads for analyte capturing. The second aptamer acted as a reporter probe. The analytical signal was generated using alkaline phosphatase (AP)-streptavidin conjugate with a chemiluminescent substrate. The aptasensor demonstrated the specificity of detection against IgG, BSA, Anti-EGFR, EGFR proteins, and limit of detection of 1 ng/mL. The authors also used this dual-aptamer detection system to evaluate VEGF165 levels in cell medium under normoxia or hypoxia conditions. However, the sensitivity of the assay was lower in comparison to ELISA.

**Figure 7:**
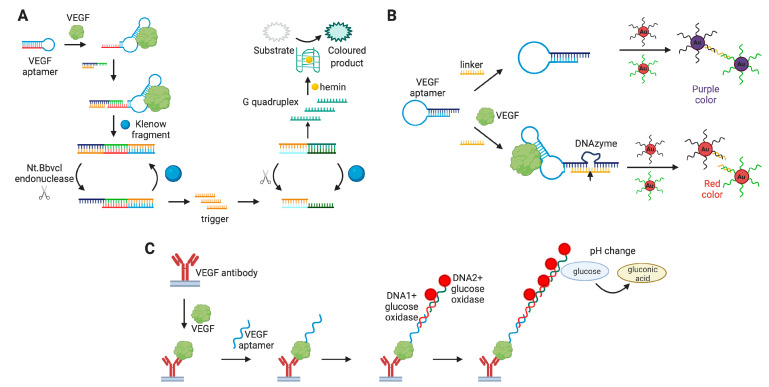
Aptasensing systems for VEGF: colorimetric aptasensor based on strand displacement amplification principle [127] (**A**), aptazyme-based system [129] (**B**), and aptasensor employing two concatemeric oligonucleotides and glucose oxidase [131] (**C**).

Several systems with signal amplification were developed to obtain VEGF colorimetric aptasensors with a lower limit of detection. For instance, Zhang et al. [127] proposed a label-free colorimetric biosensor recruiting strand displacement amplification principle (Figure 7A). A conversion of the chromogenic substrate by G-quadruplex DNAzyme in the presence of H_2_O_2_ and hemin generated the colorimetric signal. This approach provided much more sensitive detection than other VEGF colorimetric assays. Namely, the limit of detection was 0.034 pg/mL, with a dynamic range of 0.5 to 225 pg/mL and excellent specificity against HSA, transferrin, cytochrome C, and IFN-γ. The authors also verified the aptasensor’s performance in 12.5% diluted VEGF-spiked human serum samples.

C.-C. Chang et al. [128] used VEGF Apt1 DNA aptamer as a part of a bifunctional hairpin probe for engineering an aptameric amplification assay based on a combination of AuNP colorimetric detection and target-catalyzed branched DNA cascade amplification. This aptasensor demonstrated high sensitivity (the working range from 3.7 to 148 pg/mL) and specificity against BSA and IFN-γ. The authors emphasize that their assay does not require time-consuming AuNP surface modification and enzymatic amplification steps, and the detection procedure takes less than an hour.

D. Wu et al. [129] constructed an aptazyme made of a DNA aptamer and DNAzyme (Figure 7B). In the presence of the target protein, aptamer binding to VEGF induces structure reorganization of the whole aptazyme molecule, which leads to the activation of DNAzyme, cleaving the linker sequences into two fragments that fail to cross-link AuNP. Therefore, AuNPs stay dispersed, and the solution color remains red. Without VEGF, linker sequences remain intact, giving the purple color of the solution. The aptazyme system showed the specificity against HSA, BSA, and human thrombin, and the working range of 0.1–40 nM. Aptazyme-based detection was tested in 1% spiked human serum samples.

An ability of G-quadruplex forming VEGF-specific DNA aptamers to bind hemin [130] allowed for developing of the aptasensor with aptamer/hemin complex that mimics a horseradish peroxidase activity and oxide luminol by H_2_O_2_ for generating a chemiluminescent signal. This assay provided VEGF detection with a limit of 360 pg/mL. However, the sensitivity of the proposed method was much lower as compared to ELISA.

An interesting variant of the ELISA-like VEGF sandwich detection assay was proposed by H. Xu et al. [131] The assay implies using specific antibody immobilized in the microplate’s well as a capture component, and a VEGF-specific DNA aptamer (Figure 7C). An additional nucleotide sequence at the 3′-end of VEGF-aptamer served as a primer for the hybridization chain reaction with two concatemeric oligonucleotides conjugated with a glucose oxidase. In turn, the latter transforms glucose to gluconic acid and H_2_O_2_, giving the pH change, which could be monitored using a portable pH meter or pH indicator. This aptasensor provides a working range of 0.8–480 pg/mL and specificity against a number of biomarkers, namely HSA, lysozyme, thrombin, PDGF-BB, and alpha-fetoprotein. The aptasensor was also tested for the detection of VEGF in serum samples after centrifugation and 1:100 dilution. The results were comparable with those from the reference ELISA test. The same detection principle with a portable device was also reported by X. Zhu et al. [132] Their assay exploited the conjugates of concatemeric oligonucleotides with the invertase. The enzyme transforms sucrose into glucose, which is then measured by a portable glucose meter. This system gives a working range of 3–100 pg/mL and good specificity against HSA, lysozyme, and thrombin. The authors validated the assay with three different serum samples, and the obtained values were very similar to the reference ELISA results.

#### 4.1.4. Receptor Activator of Nuclear Factor Kappa-Β (RANK)

Disbalance in the RANKL/RANK/OPG system plays a fundamental role in bone resorption pathogenesis in RA and represents one of the essential mechanisms of generalized OP development [158,159]. Hyperexpression of RANKL in RA is found in many cells involved in the progression of joint inflammation: T-lymphocytes, synovial fibroblasts, and osteoclasts in the pannus zone [160,161]. Synovial macrophages in the presence of RANKL and macrophage colony-stimulating factor can differentiate into osteoclasts and stimulate osteoclastogenesis (at the stimulation by 1,25-dihydroxy vitamin D) [162,163]. An excessive RANKL concentration can be controlled by the fully human monoclonal antibody (denosumab), which binds RANKL and prevents RANK/RANKL interaction [164,165]. Changes in the RANKL/RANK/OPG system also can lead to bone metabolism disorders, such as OP, osteopetrosis, Paget’s disease, and spontaneous osteolysis [166].

Mori et al. [83] selected RNA aptamer against RANK to develop potential therapeutics for RA. The resulting 46 nt aptamer apt-1 formed a G-quadruplex structure. Both apt-1 and its 32 nt truncated version apt1 shortM4 recognized RANK but failed to inhibit RANK/RANKL interaction. Interestingly, the replacement of all pyrimidine residues by their 2′-F-modified analogs improved not only the nuclease resistance of the aptamer but also its target binding affinity. The authors also revealed that apt1 binds to other TNF receptor family proteins, namely TRAIL-R2, CD30, NGFR, and osteoprotegerin, a decoy receptor for RANK. These results suggest aptamer’s binding to some common determinant shared by the TNF receptor family.

### 4.2. Interleukins and Their Receptors

#### 4.2.1. Interleukin 17A (IL-17A) and Its Receptor (IL-17AR)

An IL-17 superfamily is a group of pro-inflammatory cytokines produced by T-lymphocytes, which sustain an autoimmune inflammation. Interleukin 17 binding to its receptor IL-17R activates a cascade of reactions that, in turn, induces the production of chemokines. Currently, IL-17 is considered to play an essential role in the pathogenesis of many rheumatic diseases. As such, the key role was proven for IL-17 in the pathogenesis of rheumatoid arthritis [167] and ankylosing spondylitis (AS) [168,169]. For AS, a high level of IL-17 associates with structural damages and radiological progression, while no such correlations were found for RA [24]. The level of IL-17 in AS is associated with the presence of enthesitis, radiographic progression with syndesmophytes formation, and the development of osteoporosis as an AS complication [24,170,171]. Moreover, in the pathogenesis of AS, IL-17 is considered together with IL-23 as a IL-17/IL-23 axis. Patients with AS showed high serum concentrations of IL-17 and IL-23 [25]. The presence of IL-17+T-cells was also shown in the facet joints of RA patients [172]. High concentrations of IL-17 activate osteoclasts and cause the OP development, while IL-23 is responsible for osteoproliferation and formation on syndesmophytes. Therefore, inhibitors of IL-17 found a broad application in AS treatment, not only lowering the disease activity but also preventing the radiological progression and the development of complications [173].

##### Aptamer-Based Inhibitors of IL-17A/IL-17RA

Ishigiro et al. [85] selected and optimized a 22 nt 2′-F-RNA aptamer Apt21-2 against human IL-17A, which also demonstrated an affinity to mouse protein, although to a lesser extent. The aptamer blocked IL-17A/IL17-RA interactions in vitro and inhibited IL17A-induced production of IL-6 in mouse and human cells. For in vivo studies, the aptamer was modified by a 40 kDa PEG at the 5′-end and inverted thymidine residue at the 3′-end. The therapeutic efficacy of this modified aptamer PEG21-2idT was evaluated on murine models of experimental autoimmune encephalomyelitis and RA. The aptamer administered intraperitoneally inhibited in a dose-dependent manner the development of arthritic or neurologic symptoms and slowed the progression of arthritis. Considering its higher affinity to human IL-17, the obtained aptamer shows a strong therapeutic potential for human autoimmune diseases. Interestingly, Apt21-2 recognized both IL-17A/A and IL-17A/F dimers, but not IL-17F/F. An alternative SELEX to IL-17A/F with counter-selection on IL-17A and IL-17F followed by sequence optimization gave the new 68 nt RNA aptamer, AptAF42dope1 [86]. The aptamer showed selective affinity to heterodimer IL-17A/F, blocked its binding to IL-17R, and inhibited IL-17A/F-induced cytokine GRO-α production in fibroblasts.

Chen et al. [84] selected the DNA aptamer RA10-6 to IL-17RA using the cell-SELEX technique with positive selection on IL-17RA positive cells and counterselection on IL-17RA-deficient cells of the same type. This approach allows obtaining aptamers that recognize the target cell-surface protein in its native form. After sequence optimization, the resulting 30 nt RA10-6 aptamer showed a high affinity to the target cells (K_D_ = 1.2 nM), recognized purified IL-17RA protein, and blocked IL-17/IL17RA interaction. Experiments on osteoarthritis mice showed that intra-articular injections of RA10-6 inhibit synovial inflammation by blocking IL-17/IL17RA-mediated IL-6 expression and did not induce systemic or immunotoxic effects. The aptamer also acted synergistically with celecoxib to inhibit IL-6 expression in synovial tissues. Therefore, aptamers blocking IL-17/IL17RA interactions show promise as potential therapeutics for osteoarthritis and probably other IL-17 rheumatic disorders.

##### Aptamer-Based IL-17A/IL-17RA Detection Assays

H. Jo et al. [133] developed an electrochemical impedimetric aptasensor to detect cells that express IL17-RA. Gold nanoparticles were electrodeposited on the working electrode and functionalized with 5′-thiol modified aptamer (purchased from Aptamer Sciences). The impedimetric detection of IL17-RA provided a working range of 10–10,000 pg/mL and sufficient specificity compared to human and bovine albumins, lysozyme, IL-5R, IL-13R, and CD166. The sensor detected neutrophil-like dHL-60 cells that express IL-17RA and neutrophils from asthma patients, thus demonstrating a potential for diagnostics for IL-17RA-related diseases.

#### 4.2.2. Interleukin 6 (IL-6) and Its Receptor (IL-6R)

IL-6 is a glycoprotein produced by lymphocytes, neutrophils, eosinophils, B-cells, fibroblasts, mast cells, endotheliocytes, synovial fibroblasts, and macrophages. In RA patients, IL-6 level significantly increases in synovial tissue, synovial fluid, and blood plasma [174]. IL-6 can activate the production of acute-phase proteins and antibodies by B-cells, chemokines’ production by endothelial cells, and expression of adhesion molecules. It also induces synovial fibroblast proliferation and activates osteoclasts. IL-6 interacts with a monomeric receptor (IL-6R), which consists of 468 amino acid residues and contains a region of 90 amino acids homological to certain domains of immunoglobulins [175,176]. The pathway of IL-6 cell signaling involves the binding of IL-6 to the α-chain of IL-6R, the coupling of IL-6/IL-6R complex to gp130, covalent homodimerization of gp130, and following cascade of intracytoplasmic phosphorylation involving JAK 1, JAK 2, TYK 2, STAT 1, and STAT 3 kinases [177,178]. The biological activity of IL-6 can be inhibited by blocking the cytokine itself, IL-6R, or gp130 molecules.

Rheumatoid arthritis is characterized by hyperproduction of IL-6, and IL-6 serum levels correlate well with combined indicators of disease activity and progression of bone tissue destruction [174]. Therefore, IL-6 represents a promising therapeutic target for RA treatment. Pathological action of IL-6 in RA is determined by the stimulation of B-cell proliferation, secretion of immunoglobulins, C-reactive protein (CRP) synthesis, and differentiation of plasmatic cells and cytotoxic T-lymphocytes [179,180]. IL-6 can participate in the development of periarticular osteoporosis and joint destruction by affecting osteoclast differentiation, increasing aggrecanase proteolytic activity, and accelerating the degradation of proteoglycans [181,182]. Nowadays, a wide range of monoclonal antibody drugs was designed targeting either IL-6 itself (sirukumab, olokizumab, clazakizumab) or its receptors (tocilizumab, sarilumab) [183]. Taking into account its influence on other cytokines, IL-6 is not considered a highly specific marker of any rheumatic disease, so its level is not routinely evaluated in clinical practice. However, the IL-6 is quite important as a marker of both inflammation and bone resorption, and its detection in clinical practice is crucial to assess the efficacy of therapy.

##### Aptamer-Based Inhibitors of IL-6/IL-6R

Aptamers capable of specific IL-6 binding also demonstrate the potential to inhibit its functional activity. Gupta et al. [87] developed two SOMAmers against IL-6 with sub-nanomolar affinities (K_D_ = 0.2 nM), SL1025, and SL1032. PEGylated versions of these aptamers blocked the interaction between IL-6 and its receptor and inhibited proliferation of tumor cell lines, such as myeloma U266B1, hepatoma HepG2, and glioma U87MG with an IC_50_ of 0.2 nM. In these model assays, the aptamers demonstrated higher efficacy than tocilizumab taken at the same concentrations. High nuclease resistance of SOMAmers, slow complex dissociation, and an ability to inhibit IL-6 signaling make them promising candidates for the development of targeted therapeutics. Further studies of their therapeutic potential included the testing of therapeutic effect of PEGylated SOMAmer SL1025 (31 nt) in a collagen-induced arthritis model in cynomolgus monkeys [184]. The aptamer formulation administered intravenously for 11 days provided a sustained reduction in plasma IL-6 levels that corresponded to the reduction of RA symptoms. Importantly, SOMAmer treatment was well tolerated in animals and did not elicit an immune response.

The research group of U. Hahn generated anti-IL6R aptamers for further use in cell delivery systems. After selection on the extracellular soluble part of the receptor as the target molecule, the resulted G-quadruplex-forming RNA aptamer AIR-3A was minimized to 19 nt [88]. The aptamer specifically bound to the IL-6R on the cell surface and was subjected to IL-6R-mediated internalization. More to the point, this short aptamer provided specific intracellular delivery of a cargo protein (streptavidin) with molecular weight ten times higher than its own. AIR-3A delivered photosensitizer chlorin-e6 for photodynamic therapy of IL-6R-positive cancer cells [185] and provided a specific internalization of gold nanoparticles [186]. After the new in vitro selection, Hahn’s group obtained even more biologically stable G-quadruplex 50 nt 2′-F-RNA aptamer FAIR-6 [89] with a sequence convergent to AIR-3A but failed to minimize it as sufficiently as AIR-3A. Both AIR-3A and FAIR-6 recognized domain 1 of IL-6R. To generate an aptamer against another aptatope, domain 3, the authors employed two SELEX targets, the soluble part of the IL-6R and domain 3 of IL-6R [90]. After selection, minimization, and post-selective replacement of all pyrimidine nucleotides by their 2′-F-analogs, they obtained the 34 nt RAID3 aptamer specific to domain 3, which was also internalized by IL-6R presenting cells. Notably, neither AIR-3A nor RAID3 interfered with IL-6-initiated signal transduction [187].

##### Aptamer-Based IL-6/IL-6R Detection Assays

**Figure 8:**
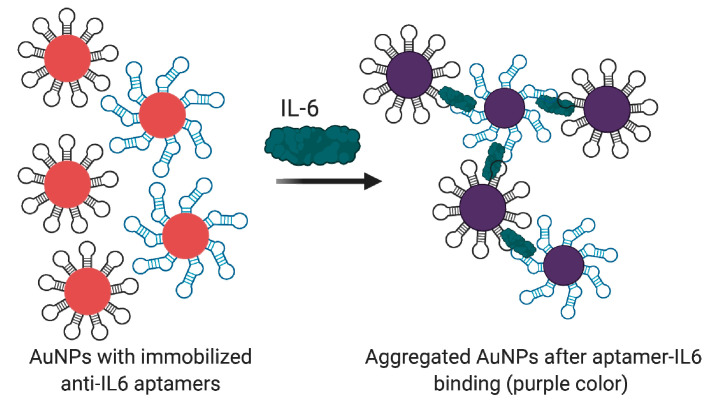
Aptasensor for IL-6 detection based on the sandwich pair of aptamers and gold nanoparticles [136].

Recently, several aptasensors were engineered for IL-6 measurement. Hao et al. presented an electrochemical graphene FET aptasensor [134]. The DNA aptamer was covalently immobilized through the terminal amino group on the graphene surface modified by pyrenebutanoic acid succinimidyl ester. The device provided fast and sensitive detection of IL-6 (with a LOD of 3.3 pg/mL) in a buffer, but its applicability in real samples was not estimated. An electrochemical impedimetric aptasensor was also developed by Tertis et al. [135] A 52-nt DNA aptamer with 3′-thio group was immobilized on the surface of modified electrode via the formation of gold-sulfur bonds and captured IL-6 from the analyzed sample. The sensor showed a linear response from 5 pg/mL to 100 ng/mL in a buffer solution and good specificity (compared to carcinoembryonic antigen, Mucin 1, Mucin 4, and Mucin 16). The authors also examined this aptasensor on serum samples diluted 1:1 and demonstrated good agreement with the results obtained by an independent chemiluminescence immunoassay. Giorgi-Coll et al. [136] used a sandwich pair of anti-murine IL-6 aptamers to create a colorimetric assay. Aptamers were immobilized on the surface of AuNPs through terminal thiol groups. The addition of IL-6 caused the aggregation of functionalized nanoparticles and subsequent red-purple color change (Figure 8). The sensor demonstrated a linear response of 3.3–125 µg/mL in a buffer. These pilot results show the principal applicability of the sensor but also clearly point to the need to optimize its sensitivity and examine the assay in more complex samples.

#### 4.2.3. Interleukin 8 (IL-8)

IL-8 is a chemokine responsible for chemotaxis of neutrophils to the inflammation area, which is produced by macrophages, lymphocytes, fibroblasts, epithelial and epidermic cells. IL-1, IL-3, TNFα, GM-CSF, and other molecules can induce IL-8 production. IL-8 exhibits prominent anti-inflammatory properties by increasing the expression of intercellular adhesion molecules and enhancing neutrophils’ adherence to endothelial cells and sub-endothelial matrix proteins. In RA, IL-8 levels in synovial fluid rise significantly due to its hyperproduction by neutrophils [188].

**Figure 9:**
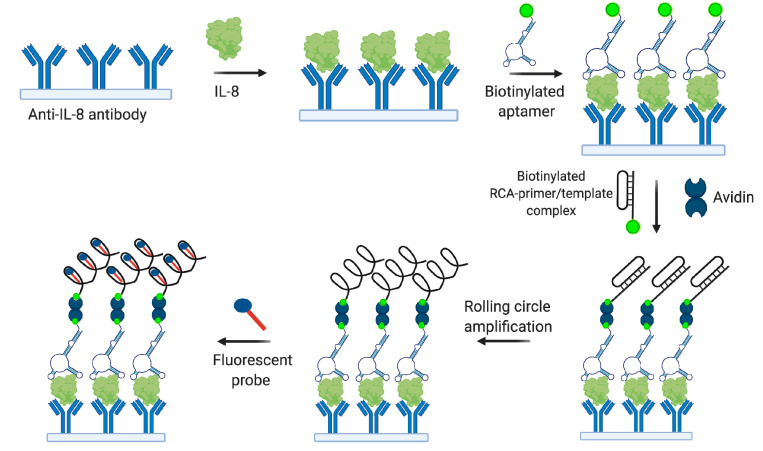
Schematic representation of the aptamer/antibody based fluorescent IL-8 detection with signal enhancement by rolling circle amplification [93].

Sung et al. [93] selected and minimized the 35 nt 2′-F-RNA aptamer 8A-35 against IL-8 with a very high binding affinity (K_D_ = 1.72 pM). The aptamer demonstrated potent IL-8 neutralizing activity and modulated multiple biological activities of IL-8, such as intracellular signaling and chemotaxis. These properties make 8A-35 a promising molecule for developing therapeutic agents against inflammatory diseases. Zhang et al. employed 8A-35 aptamer to engineer a microfluidic chip for fluorescent Il-8 detection with signal enhancement by rolling circle amplification (see Figure 9 for a detection scheme) [138]. The biosensor showed a working range of 7.5–120 pg/mL in a buffer solution and allowed analysis of the secreted IL-8 in endothelial cells.

#### 4.2.4. Interleukin 23 (IL-23)

IL-23 is a cytokine produced by different cells, such as dendritic cells, macrophages, and intestinal cells [189]. It plays a crucial role in Th17-activating and production of IL-17 [189]. It is considered that IL-23 is the main controller for NK-cells, must cells, and γ-δ T-cells that predominate in AS patients [190]. IL-23 also plays an important role in psoriasis [191] and Crohn’s disease [192]. The IL-17/IL-23 axis is important in AS, psoriasis, Crohn’s disease, and OP progression.

##### Aptamer-Based Inhibitors of IL-23

Recently, Lenn et al. [91] reported a new fully modified 60 nt RNA aptamer with 2′-O-methyl, 2′-O-methoxyethyl, and 2′-fluoro modifications and 3′-inverted terminal thymidine residue. This set of modifications seems to provide excellent nuclease resistance in biological media. Despite relatively high molecular weight (~20 kDa), the aptamer demonstrated efficient penetration through intact human skin in an ex vivo human skin model. After topical application, the aptamer accumulated in deeper skin layers at therapeutically relevant levels, and inhibited IL-23 signaling, thus representing a potential therapeutic agent for the treatment of psoriasis.

#### 4.2.5. Other Interleukins

##### Aptamer-Based Inhibitors of Interleukins

Kim et al. [92] generated a 90 nt RNA aptamer AC3-3 against IL-32. The aptamer antagonized the functional activity of IL-32 and inhibited IL-32-dependent expression of TNFα in human lung carcinoma cells. As a selective antagonist of IL-32, the AC3-3 aptamer can be considered as a potential anti-inflammatory therapeutic agent.

Ren et al. selected, truncated to 22 nt, and thoroughly characterized the SOMAmer SL1067 against IL-1α [94]. The aptamer selectively bound IL-1α but not IL-1β and possessed a very compact spatial structure comprising an unknown G-quadruplex form. The ability to inhibit IL-1α-mediated secretion of IL-6 and IL-8 in HS27 and HUVEC cell lines makes the SL1067 aptamer a useful foundation for developing new therapeutics targeting IL-1α and related cytokines.

##### Aptamer-Based Detection Assays

A rapid, washing-free colorimetric aptasensor for detecting the soluble IL-2Rα was developed on the basis of the cognate sIL-2Rα-specific C5-dU-naphthyl-modified DNA aptamer [137]. The aptasensor employs the peroxidase mimetic activity of the AuNP. First, the aptamer molecules non-covalently bind to the surface of AuNP through the DNA adsorption mechanism. The increased negative net charge attracts more ortho-phenylenediamine (oPD), a positively charged peroxidase chromogenic substrate. After the addition of sIL-2Rα, the aptamers desorb from nanoparticles, which weakens the oPD, thus decreasing the colorimetric signal. The sensor provided the working range of 25–2500 ng/mL and showed high specificity in experiments with other immunity-associated proteins such as IL-5Rα, IL-13Rα_2_, IL-17Rα, and CD166. The feasibility of the aptasensor for detection in clinical samples was validated in sIL-2Rα-spiked diluted human sera. The working range in model serum samples was the same as in the buffer. The whole detection process is simple, relatively inexpensive, and fast (it takes approximately 25 min). Therefore, this method seems very promising for diagnostics of inflammatory diseases.

### 4.3. Other Specific Markers

#### 4.3.1. WNT Pathway

The WNT signaling pathway plays a key role in cell proliferation and determines the pathogenesis of various autoimmune disorders [193]. WNT activation can proceed through a canonical pathway involving β-catenin, which indirectly stimulates NF-κB activation and production of anti-inflammatory cytokines (IL-1B, TNFα, IL-6) [194], or through the non-canonical pathway without β-catenin. WNT signaling results in the production of anti-inflammatory cytokines, activation of osteoblasts by enhancing osteoprotegerin expression, and a decrease of RANKL expression [195]. Sclerostin and DKK-1 inhibit WNT signaling and participate in the pathogenesis of AS, affecting bone remodeling with syndesmophytes formation [196,197]. The increase of WNT signaling antagonist expression promotes the development of osteoporosis. WNT signaling components are now considered as therapeutic targets for monoclonal antibodies. For instance, romosozumab, a sclerostin-blocking monoclonal antibody, showed good efficacy and safety in OP therapy [198,199,200,201].

##### Aptamer-Based Inhibitors of WNT Proteins

Sclerostin (Sn) is mostly specific for bone tissue, secreted mainly by osteocytes, with the highest level of this protein in canaliculi and lacunae of osteocytes [193,202]. To a lesser extent, it can be produced by vascular cells [203]. Sclerostin binds to co-receptors LRP5 and LRP6 on the osteocyte surface and prevents the formation of the WNT-FZD-LRP5 complex, thus breaking off WNT signaling, hindering osteoblastogenesis and bone tissue formation. Studies of hereditary diseases associated with SOST gene mutations, such as sclerosteosis and van Buchem disease, allow tracing out the influence of Sn on osteoblastogenesis [204,205]. Expression of sclerostin in osteocytes is regulated by hormones involved in bone tissue metabolism: parathyroid hormone (PTH), calcitonin, and glucocorticoids [193,202]. RA patients demonstrated higher Sn levels in serum, significantly correlating with disease activity indicators and inflammation markers, but not with bone destruction [206]. Aside from the loss of bone mass, RA is characterized by accelerated atherosclerosis, previously assigned to anti-inflammatory cytokines and traditional risk factors. However, a mutation in LRP5, the co-receptor target of Sn, causes early damaging of coronary arteries and severe osteoporosis [207]. Therefore, Sn is possibly involved in joints and cardiovascular diseases and can participate in RA pathogenesis.

Shum et al. [96] performed a selection of sclerostin-binding 30 nt DNA aptamer Scl 2, which formed a parallel G-quadruplex structure, and supplied it with 3′-inverted thymidine to enhance biological stability. The aptamer inhibited sclerostin’s antagonistic effect on WNT signaling in MC3T3 osteoblasts, showing a dose-response with an IC_50_ of 900 nM. Therefore, this aptamer can be considered as a potential sclerostin-inhibiting therapeutic.

##### Aptamer-Based Detection Assays for WNT Proteins

Dickkopf-1 is a soluble inhibitor of the WNT pathway, playing a key role in the regulation of bone metabolism. Its levels defined the development of erosion or formation of new bone tissue in a model of inflammatory arthropathy [208]. DKK-1 affects the AS pathogenesis: AS patients showed a high level of DKK-1, which further increased during the treatment by TNFα inhibitors [209]. Furthermore, the functional activity of DKK-1 determines the development of syndesmophytes and sacroiliitis. A strong connection was revealed between the DKK-1 and CRP levels and the number of syndesmophytes [210]. DKK-1 also affects the development of both primary and secondary osteoporosis. Serum levels of DKK-1 vary during anti-OP treatment with different drugs [211,212]. Therefore, anti-DKK-1 monoclonal antibodies represent promising therapeutics for OP treatment [213]. Simultaneously, TNFα does not directly regulate DKK-1 production, so anti-TNFα therapy does not affect radiological AS progression [214].

Zhou et al. [95] developed a 39 nt DNA aptamer TD10 for DKK-1 and employed the aptamer/antibody sandwich pair (with the aptamer as a capture component) in an ELISA-like colorimetric microplate assay. The aptamer/antibody construction demonstrated analytical performance close to conventional ELISA with a working range of 62.5-4000 pg/mL. Quantification of the large set of sera samples (diluted 1:10) were also well correlated with the conventional ELISA, which points to the potential of this assay for clinical practice.

#### 4.3.2. Connective Tissue Growth Factor (CTGF)

Connective tissue growth factor (CTGF or CCN2), an extracellular matrix regulatory protein of the CCN family, represents one of the key regulators of fibrotic scarring in health and disease [215]. CTGF synthesis is stimulated mostly by transforming growth factor β (TGFβ), causing the production of type 1 collagen and fibronectin, proliferation of fibroblasts and osteoblasts, and repair of lesions. Alongside other factors, CTGF affects many fibrosis-associated disorders, such as systemic scleroderma, which was proved on animal models [216]. The level of CTGF expression increases with fibrosis progression, and serum levels of CTGF correlate with the extents of skin involvement and pulmonary lesions in scleroderma [217]. Direct blocking of CTGF in murine skin fibroblasts by FG-3019 antibody (pamrevlumab) decreased inflammation, fibrosis and vascular lesions provoked by angiotensin II [218]. Skin fibroblasts of scleroderma patients show a decreased level of PTEN, which limits CTGF activity [219]. This can also prove the potential of CTGF as a therapeutic marker in the regulation of fibrosis progression.

##### Aptamer-Based CTGF Detection Assays

**Figure 10:**
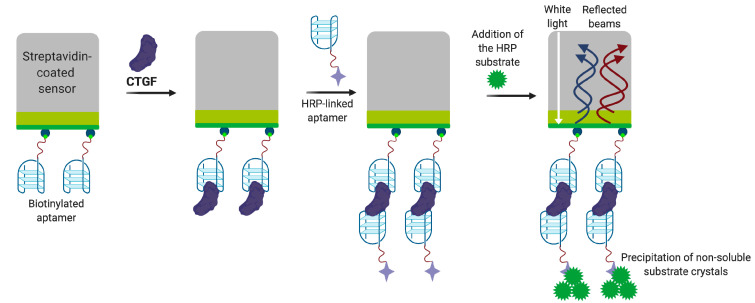
CTGF aptasensing system based on biolayer interferometry and enzyme-linked aptamer sandwich assay [97]. The precipitation of non-soluble substrate crystals at the final step causes a significant spectral shift and amplifies the detection signal.

Gao et al. [97] selected a DNA aptamer against CTGF, using a counter-selection on several targets such as thrombin, TNF-α, LCN 1, LCN 2, and SEMA 3A to improve the specificity. The resulting aptamer APT1 was truncated to a 21 nt sequence, which formed an antiparallel G-quadruplex. The introduction of LNA nucleotides into single-stranded regions connecting G-tetrads further stabilized the structure and improved the nuclease resistance of modified APT1M6TL aptamer. To test the possibility of using the aptamer in diagnostic assays, the authors engineered a quite unusual ELISA-like sandwich assay on the biolayer interferometry (BLI) platform, which provided the amplification of analytical signal (Figure 10). Interestingly, the LNA-modified aptamer acted as a capture component, and a non-modified aptamer of the same sequence was chosen as the reporting component of the sandwich. The working range of the assay was 1.1–112 ng/mL, and the system showed good reproducibility and stability for the analysis of diluted (1:10) spiked serum and urine samples. Therefore, the obtained aptamer looks promising for engineering CTGF diagnostic assays in biological samples.

A series of CTGF-binding 39 nt DNA aptamers with relatively high affinities in the nanomolar range were also selected in [98] but have not yet been employed for diagnostic or therapeutic purposes.

#### 4.3.3. Osteopontin

Osteopontin (OPN) is a glycoprotein with cytokine-like properties that presents in bone and teeth and takes part in bone metabolism [220] and bone mineralization. It has a high affinity to calcium and could bind hydroxyapatites and cause differentiation of osteoclasts [221,222]. It was established that OPN-deficiency causes a decrease in fracture toughness [223]. OPN also seems to take part in pathogenesis of cancer, fibrosis, and neurology disorders [224,225,226].

##### Aptamer-Based OPN Inhibitors

Mi et al. [99] selected a 40 nt 2′-F-RNA aptamer OPN-R3 specific to both human and mouse osteopontins. The aptamer inhibited osteopontin binding with its cell surface receptors CD44 and α_v_β_3_-integrin in cell culture assays. For in vivo studies, the aptamer was modified by 3′-inverted deoxythymidine, 5′-cholesterol, and 2′-O-methyl pyrimidine nucleotides. It seems somewhat surprising that the replacement of 2′-fluoro atoms by –OCH_3_ groups did not significantly decrease the aptamer’s binding affinity. After injection in the tail vein, the modified aptamer showed anti-tumor activity in mice with xenograft tumors. Although this model is not directly related to rheumatic diseases, the results clearly show that the aptamer can inhibit osteopontin’s signaling in vivo after systemic delivery.

##### Aptamer-Based OPN Detection Assays

**Figure 11:**
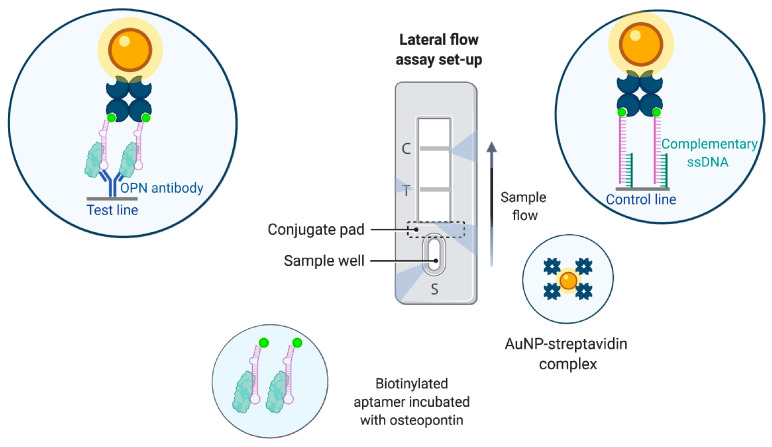
Lateral flow aptasensor for osteopontin detection based on aptamer/antibody sandwich pair and gold nanoparticles [139].

The DNA aptamer against human osteopontin was employed in [139] to develop lateral flow strips for fast and sensitive osteopontin detection (Figure 11). The biotinylated aptamer provided pre-capture of the target from the sample, the specific antibody was immobilized on the test line for second specific target identification, and streptavidin-modified AuNP were responsible for color detection. The resulting red zone was seen by the naked eye and evaluated semi-quantitatively by the strip reader. The use of these test strips allowed OPN detecting in the range of 10–500 ng/mL. The sensor was also successfully tested on diluted spiked and clinical serum samples.

##### DEK Protein

DEK is an oncoprotein with cellular functions including (but not limited to) activities in modifying chromatin structure, transcription and DNA repair regulation, RNA splicing, and inflammation [227]. Increased levels of DEK expression were revealed in tumors of different types. DEK is also secreted into intracellular space by activated neutrophils participating in the generation of neutrophil extracellular traps (NET). Anti-DEK antibodies were found in blood sera of patients with autoimmune disorders [228].

##### Aptamer-Based DEK Inhibitors

Mor-Vakhin et al. [100] generated a 41 nt DNA aptamer DTA-64 for DEK protein, aiming to obtain the therapeutic agent for inflammatory arthritis. The aptamer injected into the knee joint of mice with zymosan-induced inflammatory arthritis reduced the inflammatory cell migration and levels of IL-1β and IL-6. Therefore, targeting DEK with aptamer therapy is a potentially useful approach to treating arthritis, although further optimization of the aptamer’s formulation is necessary for subsequent in vivo studies.

#### 4.3.4. Visfatin

Visfatin, also known as nicotinamide phosphoribosyltransferase (NAMPT), catalyzes the rate-limiting first step of NAD synthesis from nicotinamide. Its extracellular activity is associated with participation in the inflammation by affecting macrophages and interaction with TLR4. Thus, visfatin represents one of the soluble factors with damage-associated molecular patterns (DAMP)-like activity [229]. The increase of the NAMPT level and its correlation with disease activity were shown for psoriasis, RA, osteoarthritis, and inflammatory bowel diseases. In the case of RA, visfatin is considered as a therapeutic target [230].

##### Aptamer-Based Visfatin Detection Assays

Park et al. [101] selected a visfatin-recognizing DNA aptamer using counter-selection against other adipokines (adiponectin and retinol-binding protein 4) and HSA. The resulting aptamer 19 was then employed as a bio-specific element of an electrochemical capacitive biosensor based on non-Faradaic impedance spectroscopy. This detection method provided a specific concentration-dependent signal with a working range of 1-50 ng/mL in a buffer solution. The sensor also showed a dose-dependent, specific capacity response in visfatin-spiked serum samples (diluted 1:5).

#### 4.3.5. Matrix Metalloproteinase 9 (MMP-9)

Matrix metalloproteinase 9 (also referred to as gelatinase-B) secretes as a zymogen with a molecular weight of 92 kDa. Its substrates include denatured type 1 collagen (gelatin), native collagens of types IV, V, VII, X, and XI, fibrinogen, vitronectin, IL-1, and entactin, which joins laminin and type IV collagen [231]. MMP-9 is produced by different cells, including keratinocytes, monocytes, tissue macrophages, polymorphonuclear leukocytes, as well as various malignant cells. MMP-9 cleaves the denatured type IV collagen, the main component of basal membranes, enabling the invasion of immune cells (including T-cells) to the damaged tissue. Increased MMP-9 levels are found mostly not in the serum but in synovia and synovial fluid in arthritis patients and in unstable atherosclerotic plaques in patients with atherosclerosis. The treatment decreasing MMP-9 level or activity can be a therapeutic variant for autoimmune disorders. Intravenous γ-globulins and steroids can decrease the amount of secreted MMP-9 and its expression [232]. So far, no targeted drugs are known for inhibiting MMP-9 activity.

##### Aptamer-Based MMP-9 Detection Assays

Da Rocha Gomes et al. [102] developed a 2′-F-pyrimidine RNA aptamer F3B against human MMP-9, truncated it to 36 nt, and replaced purine nucleotides with their 2′-O-methyl analogs. The resulting modified aptamer F3Bomf possessed high binding activity and selectivity and distinguished MMP-9 from matrix metalloproteinases MMP-2 and MMP-7. The authors employed the ^99m^Tc-containing derivative of F3Bomf for in vivo tumor imaging.

The F3Bomf 2′-F-RNA aptamer was also used for engineering a piezoelectric biosensor based on the quartz crystal microbalance detection principle [103]. An additional selection of DNA aptamer against the MMP-9 catalytic domain [103] provided a second MMP-binding aptamer, which possessed a G-quadruplex secondary structure and did not compete with F3B for binding to MMP-9. The use of an aptamer sandwich pair allowed for specific and sensitive assay with a working range from 92 pg/mL to 230 ng/mL in a buffer solution. The sensor was also successfully tested in MMP-spiked diluted (1:100) human serum. Of note, pre-treatment of serum samples by magnetic beads for removal of immunoglobulins improved the analytical performance of the sensor.

#### 4.3.6. C-Terminal Telopeptide (CTX-I)

C-terminal telopeptide of collagen type I (CTX-I) is a biomarker of bone metabolism. CTX-I peptide undergoes further degradation with the formation of α- and β-isomeric octapeptides (α- and β-CrossLaps). The International Osteoporosis Fond (IOF) and International Federation of Clinical Chemistry and Laboratory Medicine (IFCC) recommended CTX-I as a reference marker for OP management. It is now actively used in routine medical and scientific practice to monitor OP therapy and evaluate its efficacy [233]. The decrease of β-CrossLaps concentration by >25% from the starting level in 3–6 months from the beginning of therapy points to antiresorptive treatment efficacy. The β-CrossLaps level increases with age. High levels of C-telopeptides were found in women with low BMD. At the same time, OP patients receiving antiresorptive therapy demonstrated a reliable decrease in those markers [234,235]. Different clinical and pre-clinical studies showed enhanced collagen degradation in RA due to the high proteolytic activity of CTX-1 [236].

##### Aptamer-Based CTX-I Detection Assays

Bruno et al. [104] selected DNA aptamers against CTX-I and performed a thorough post-selective design supported by structure modeling to obtain short hairpin structures suitable for fluorescence molecular beacon assay. Fluorophore and quencher introduced to the opposite ends of the aptamer provided fluorescence signaling in a concentration-dependent manner. In the buffer solution, it gave a limit of detection of 1 ng/mL with a good specificity compared to several other bone markers. The authors intended to use this aptasensor to quantitate the CTX-I in urine samples for fast and easy bone resorption monitoring. However, it turned out that urea and creatinine dramatically destabilize the hairpin structure, and chromatographic pre-processing of the samples is required before the fluorescence analysis. Therefore, when designing aptasensor constructs for urine analysis, the researchers should keep in mind the impact of denaturing amines and urea.

#### 4.3.7. Human Neutrophil Elastase (HNE)

HNE is a serine protease that is contained in neutrophils granules and acts in pathogen destructions, such as killing bacteria by oxygen metabolites with phagolysosomes and the NADPH system [237]. It is expected that HNE can regulate inflammation by the degradation of some cytokines such as TNFα or their predecessors [238]. HNE is involved in the pathogenesis of ANCA-associated vasculitides [239,240,241]. In such diseases, neutrophils are activated by binding with ANCA and produce reactive oxygen species and neutrophil extracellular traps that deposit in vessels and cause necrotizing glomerulonephritis [242].

Lin et al. [105] selected, optimized, and characterized a 44 nt G-quadruplex DNA aptamer DNA I against HNE. Interestingly, a conjugation of the aptamer with tetrapeptide Ala-Ala-Pro-Val, a weak inhibitor of HNE, resulted in nearly five orders of magnitude more potent competitive inhibition than by the peptide alone. It could be suggested that the aptamer attached to the peptide through 3′-thiol group tethered to three 18-carbon linkers allows for tight binding and precise positioning of the peptide near the substrate binding center.

##### Aptamer-Based HNE Detection Assays

**Figure 12:**
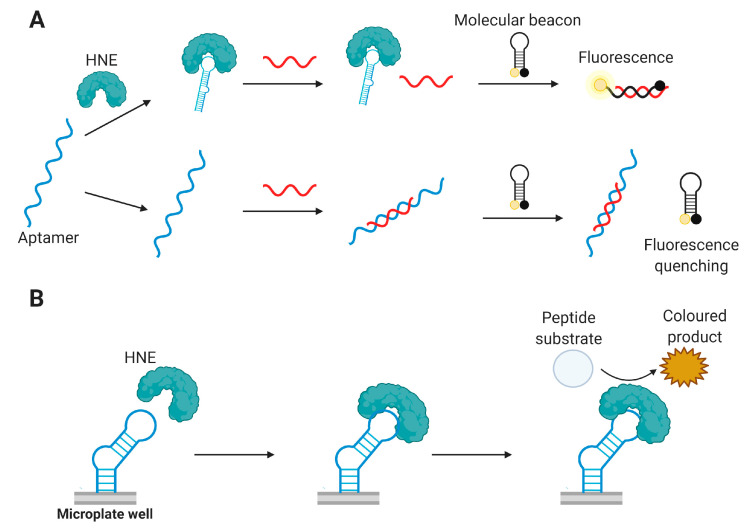
Aptasensors for HNE detection: fluorescent aptasensor employing molecular beacon (**A**) [140] and colorimetric ELISA-like system with a chromogenic peptide substrate [141] (**B**).

The aptamer DNA I found its further application as a biospecific element of aptasensors for HNE detection. He et al. [140] developed a fluorescent aptasensor which involved a molecular beacon and an auxiliary DNA oligonucleotide (Figure 12A). The sensor showed good specificity for HNE compared with HSA, IgG, and IgE, and a working range of 31.2–3100 ng/mL in a buffer solution.

Cheng et al. [141] employed an intrinsic enzymatic activity of HNE to obtain a colorimetric signal. An anti-HNE aptamer was immobilized on magnetic beads or microplate, then captured HNE catalyzed the conversion the chromogenic peptide substrate to the coloured product (Figure 12B). During the specificity test, other proteins (e.g., trypsin, proteinase K, chymotrypsin, thrombin, lysozyme) did not influence the assays. The working range for ELISA-like microplate format of the assay was 0.06–15 ng/mL in a buffer solution. However, in spiked diluted serum the presence of HNE inhibitors, such as alpha-1-antitrypsin, significantly lowered the performance of the aptasensor. To overcome the problem, the authors pre-heated the serum before spiking, but this protocol can hardly be recommended for analyzing real serum samples.

Bai et al. [142] proposed using a fluorescently labeled aptamer to detect HNE by capillary electrophoresis (CE) with a laser-induced fluorescence assay. The electrophoretic mobility of HNE/aptamer differed very well from the unbound aptamer in CE separation. The aptamer was labeled by tetramethylrhodamine attached to the thymine residue in the 40th position of the oligonucleotide chain. The assay demonstrated the working range of 15.6–15 600 ng/mL and good specificity (IgG, hemoglobin, thrombin, and PDGF-BB as controls). The authors also sufficiently tested the assay for HNE detection in 1:100 diluted spiked serum samples with a limit of detection of 800 ng/mL.

#### 4.3.8. Hepatocyte Growth Factor (HGF)

Hepatocyte growth factor (HGF) is a cytokine that participates in embryogenesis, histogenesis, cancerogenesis, and in repair processes. HGF induces processes of proliferation and regeneration by interacting with its tyrosine kinase receptor c-MET [243] and regulates bone metabolism processes through expression in osteoblasts and osteoclasts [244]. HGF also affects the pathogenesis of rheumatic diseases. In particular, HGF expression was demonstrated in the synovium of patients with RA and osteoarthritis [245]. Sugiura et al. [246] observed the presence of HGF in striated muscles and proved the role of this cytokine in polymyositis progression. They also described an increase of HGF expression on myoblasts after dexamethasone treatment and suggested that a combination of HGF and dexamethasone can be more effective in polymyositis therapy. The role of HGF was also proven in the progression of AS. L. Torres et al. [247] demonstrated higher levels of HGF in the serum of AS patients compared to healthy donors, which directly correlated with the disease activity and inversely correlated with the level of BMD. This observation demonstrates the importance of this cytokine in diagnostics and treatment of AS and osteoporosis as a frequent AS complication.

##### Aptamer-Based HGF Inhibitors

Saito et al. [106] generated two 60 nt DNA aptamers for HGF—a hairpin H38-15 and a G-quadruplex H38-21—both with a nanomolar binding affinity. The aptamers inhibited the functional activity of HGF in cell culture assays involving KP-3 and HUVEC cells. Considering their high affinity and inhibiting properties, these aptamers seem promising for diagnostic and therapeutic use.

#### 4.3.9. Leptin (Lp)

Leptin is a non-glycosylated 16 kDa protein of cytokines superfamily type 1, mostly produced by adipocytes. Aside from its biological function of fat accumulation and energetic consumption, leptin also affects the immune system regulation for innate and adaptive immunities. At the same time, it directly participates in the development of autoimmune diseases [248]. In patients with SLE, higher levels of Lp were found in comparison to remission patients but this does not correlate with the severity of proteinuria in lupus nephritis [249]. The serum level of Lp inversely correlates with circulating Treg lymphocytes, and thus in SLE, Lp can disrupt the regulatory mechanism of adaptive immunity [59]. A positive correlation between CRP and Lp levels was shown [250]. Patients with RA demonstrated increased Lp levels [251]; those with positive ACCP and obesity exhibited higher levels of Lp [250]. In synovial fluid, higher Lp levels are also noticed in comparison to osteoarthritis patients [252,253]. The correlation between Lp and cardiovascular risks in RA was already established [254].

Ashley et al. [107] reported the selection of anti-leptin DNA aptamers by a CE-SELEX. The obtained 40 nt aptamers Lep1, Lep2, Lep3, and Lep4 demonstrated high-nanomolar binding affinities with the best affinity of 410 nM for Lep2. Further studies on these aptamers have not yet been published.

#### 4.3.10. Oncostatin M (OSM)

OSM is a member of the IL-6-cytokine family that regulates immune reactions and plays an essential role in endothelial dysfunction and fibrosis [255,256]. An increase in OSM production influences the progression of a wide range of pathologies, including atherosclerosis, psoriasis, and many kinds of cancer [257]. Recently, the role of OSM in inflammatory bowel disorder was demonstrated, and the increase in OSM levels correlated with disease severity [258]. In RA, OSM is excessively expressed in synovial fluid and the tissues with active inflammation [259,260]. Therefore, OSM stimulates intra-articular inflammation and destruction of cartilage tissue, which was proved in studies on murine arthritis models [261]. Simultaneously, OSM can exhibit pleiotropic effect and express functions of both pro- and anti-inflammatory cytokine, dependent on cell type and microenvironment. Thus, this protein plays a complicated role in RA, mostly dependent on cell microenvironment [262].

##### Aptamer-Based OSM Inhibitors

Rhodes et al. [108] isolated 2′-F-Py RNA aptamers against OSM to investigate its role in inflammatory disorders. The resulting 33 nt aptamer ADR58 with a low-nanomolar affinity (K_D_ = 7 nM) was subjected to post-selective modification, with the replacement of all purine residues by their 2′-O-methyl analogs. In cell culture assays, ADR58 blocked the binding of human OSM to the gp130 receptor in a dose-dependent manner, therefore representing a highly potent functional antagonist of human OSM.

## 5. Conclusions and Future Directions

Nucleic acid aptamers established themselves as affine and specific tools for biomedical applications. A vast number of aptamer-based targeted therapeutics and bioanalytical assays have been proposed to the moment. While studies in this field focus mostly on aptamers’ applications in the fields of infectious, cardiovascular, and malignant diseases, the potential of aptamers for treatment and diagnostics of rheumatic disorders is also very promising. As it is seen from the works referenced above, aptamer-based targeted drugs and bioanalytical assays already found numerous applications in rheumatology research studies. Different DNA and RNA aptamers and their modified analogs have been generated against protein biomarkers related to rheumatic diseases, from general markers such as C-reactive protein, TNFα, interleukins, and their receptors, to more specific proteins such as members of the WNT signaling pathway. Some of these aptamers demonstrated promising potential as specific therapeutics, inhibiting target proteins’ functional activities in cell assays and animal models. Other aptamers showed themselves as biospecific recognizing elements for the engineering of sensitive and specific aptasensors. In many cases, the use of such aptasensors for analysis of clinical samples faces the problem of interfering components (such as proteins and amines) and therefore requires the pre-processing of the probes. This issue has to be addressed for the routine use of aptamer-based diagnostic assays. Otherwise, the examples of aptasensors effectively employed for biomarker detection in clinical probes with no other pre-treatment as minimal dilution strongly suggest that this problem can be successfully overcome. Further efforts in this field should be concentrated on broadening the variety of aptamers for biomarkers, in-depth structural studies of these aptamers and their protein complexes, engineering of aptasensors suitable for routine lab diagnostics, and developing and testing therapeutic aptamers.

It should be emphasized that many aptamer-based diagnostic sandwich assays reviewed above rely on the use of aptamer/antibody sandwich pairs. In our opinion, the replacement of only one antibody in the sandwich by the aptamer counterpart improves the cost-efficiency and, probably, the specificity of the assay. Nevertheless, such combined aptamer/antibody pairs are still prone to problems of reliability and long-term reproducibility brought by the antibody component. Therefore, we should pay more attention to the generation of aptamer/aptamer sandwich pairs, which are now available only for a limited number of biomarkers. For instance, universal approaches to the selection of such pairs have been proposed by Ochsner et al. [263]

We would also like to mention here an emerging trend in the aptamers’ engineering for biomedicine. Very recently, it was proposed to generate aptamers against therapeutic monoclonal antibodies [264,265]. Thanks to their unique specificity and ability to recognize a certain spatial conformation of the protein, aptamers represent precise molecular tools for quality control of therapeutic antibodies. In particular, aptamers may provide a possibility to compare originators and biosimilar biopharmaceuticals, to monitor lot-to-lot consistency between different batches of the same antibodies and to assess the product quality during transportation and storage. For example, DNA aptamers against anti-CD20 antibody rituximab detected structural differences upon thermal denaturation and demonstrated the high similarity of rituximab originator and biosimilars, and changes for all the lots of a copy product [265,266]. DNA aptamers against anti-HER2 trastuzumab showed the potential to detect the target in complex samples and distinguished native antibodies from heat-treated ones [264]. As the treatment of many rheumatic disorders largely relies on the use of targeted monoclonal antibodies, the development of cognate aptamers and aptamer-based tools for their quality monitoring represents, in our opinion, an up-and-coming trend in the field. Until now, no aptamers have been reported against biopharmaceuticals used in rheumatology, but it is undoubtedly only a question of time.

To summarize, aptamers represent readily available, versatile, and effective molecular tools for therapeutic and diagnostic tasks in rheumatology. It is no doubt that greater diversity of aptamers for proteins associated with rheumatic disorders, as well as aptamer-based platforms, will appear in the near future.

## Figures and Tables

**Table 1:** Aptamers for protein biomarkers associated with rheumatic disorders.

Target	Aptamer, Type, Length	Sequence, 5′-> 3′	**Binding Affinity (K_D_)**	**Ref.**
CRP	RNA 44-mer (RNA, 44 nt)	GCCUGUAAGGUGGUCGGUGUGGCGAGUGUGUUAGGAGAGAUUGC	-	[73]
	CRP1-1 RNA, 104 nt	GGGCGAAUUCGGGACUUCGAUCCGUAGUACCCACCAGGCAUACACCAGCACGCGGAGCCAAGGAAAAAUAGUAAACUAGCACUCAGUGCUCGUAUGCGGAAGCU	2.3 nM	[74]
	Clone 1 DNA, 71 nt	GGCAGGAAGACAAACACGATGGGGGGTATGATTTGATGTGGTTGTTGCATGATCGTGGTCTGTGGTGCTGT	3.51 nM	[75]
	6th-62-40, DNA, 52 nt	CGAAGGGGATTCGAGGGGTGATTGCGTGCTCCATTTGGTGTTTTTTTTTTTT	16.2 nM	[76]
	CRP-80-17 DNA, 79 nt	AGCAGCACAGAGGTCAGATGCCCCGCGGGTCGGCTTGCCGTTCCGTTCGGCGCTTCCCCCCTATGCGTGCTACCGTGAA	3.9 nM	[77]
TNFα	aptTNF-α DNA, 41 nt	GCGCCACTACAGGGGAGCTGCCATTCGAATAGGTGGGCCGC	8 nM	[78]
	VR11 DNA, 25 nt	TGGTGGATGGCGCAGTCGGCGACAA	7 nM	[79]
	T3.11.7, 2′-NH_2_- RNA, 28 nt	*GGAG*U*A*UCU*GA*U*GA*C*AA*UUC*GGAG*CUCC	-	[80]
	T1-4, DNA, 49 nt	TCCGATCGGTATATCCGTCGGATTTTTTTTTTGGTCACTGCATGTGACC	67 nM	[81]
VEGF	VEGF Apt 1 DNA, 24 nt	GTGGGGGTGGACGGGCCGGGTAGA	-	[82]
	VEGF Apt 2 DNA, 26 nt	CAATTGGGCCCGTCCGTATGGTGGGT	-	[82]
RANK	apt1 2′-F-RNA, 46 nt	A*C*GGA*UUC*G*UC*G*U*A*U*GGG*U*GGGA*UC*GGGAAGGG*CU*A*C*GAA*C*G*CC*G*U*	0.6 μM	[83]
IL-17RA	RA10-6 DNA, 30 nt	CTTGGATCACCATAGTCGCTAGTCGAGGCT	1.2 nM	[84]
IL-17A	Apt21-2 2′-F-RNA, 33 nt	GG*UCU*AG*CC*GGAGGAG*UC*AG*U*AA*UC*GG*U*AGA*CC*	48.5 nM	[85]
IL-17A/F	AptAF42-dope1 2′-F-RNA, 68 nt	GGG*CU*AG*CU*GA*UC*GUA*CC*AG*U*AG*C*G*U*GG*CCU*GGGGGG*CCU*AG*UC*G*U*G*C*GA*U*A*CU*AA*C*AG*CU*AA*C*A*CCC*	-	[86]
IL-6	SL1025 SOMAmer, 31 nt	GGCAG*BnBnPe*GG*Nap*A*BnBn*AACACG*BnBn*AAG*Bn*CG*Bn*GG	0.19 nM	[87]
IL-6R	AIR-3A RNA, 19 nt,	GGGGAGGCUGUGGUGAGGG	60 nM	[88]
	FAIR-6 2′-F-RNA, 50 nt	G*U*AAG*U*AG*U*G*U*AGG*CU*G*U*GGGAG*UU*A*U*AGGGG*U*GGA*U*G*U*GGAG*U*GGGG*U*G	41 nM	[89]
	RAID3 2′-F-RNA, 34 nt	GGGAGAA*CU*G*U*GGGAG*U*GGAGGG*U*GGA*U*GG*UUCU*	43 nM	[90]
IL-23	RNA (mRfY) 60 nt	*AGGGAAAUCAGGCUUUAUCGGCGCCGCUCCCUGUGCCAUCGUCCGAGAGUAGGUAGUCUG*	-	[91]
IL-32	AC3-3 RNA, 90 nt	GGGUUCACUGCAGACUUGACGAAGCUUCCGGAGAGAAGGGUCAAAGUUGUGCGGGAGUGUGUUGUGGAAUGGAUCCACAUCUACGAAUUC	78 nM	[92]
IL-8	8A-3 52′-F-RNA, 35 nt	GGGGG*CUU*A*UC*A*UUCC*A*UUU*AG*U*G*UU*A*U*GA*U*AA*CC*	1.72 pM	[93]
IL-1α	SL1067 SOMAmer, 22 nt	CG*Nap*GAG*NapNap*A*Nap*GGG*NapNap*AGAG*Nap*CG	7.3 nM	[94]
DKK1	TD10 DNA, 39 nt	CATATGATTAGGCTGTAACGGGGCTAGGCGGGGATCATT	25 nM	[95]
Sclerostin	Scl DNA, 30 nt	TTGCGCGTTAATTGGGGGGGTGGGTGGGTT	0.67 μM	[96]
CTGF	APT1M6T DNA	not reported	1.1 nM	[97]
	C-ap11 DNA, 39 nt	GGACAAGAATCACCGCTCCCCGTACAGGAGGCATACAGA	7.4 nM	[98]
Osteopontin	OPN-R3 2′-F-RNA, 40 nt	CGG*CC*A*C*AGAA*U*GAAAAA*CCUC*A*UC*GA*U*G*UU*G*C*A*U*AG*UU*G	18 nM	[99]
DEK	DTA 64 DNA, 41 nt	GGGGTTAAATATTCCCACATTGCCTGCGCCAGTACAAATAG	-	[100]
Visfatin	apt№19 DNA, 75 nt	ATACCAGCTTATTCAATTGGGCAGGACAGGTGTCGGCTTGATAGGCTGGGTGTGTGTAGATAGTAAGTGCAATCT	72 nM	[101]
MMP9	F3Bomf 2′-F-RNA, 36 nt	*U*G*CC*AAA*C*G*C*G*UCCCCUUU*G*CCC*GG*CCUCC*G*CC*G*C*A	20 nM	[102]
	8F14A, DNA, 30 nt	TCGTATGGCACGGGGTTGGTGTTGGGTTGG	-	[103]
CTxI	CTx 2R-2h DNA, 72 nt	ATCCGTCACACCTGCTCTAGACGAATATTGTATCCTCATTAGATCAAAAACGGGTGGTGTTGGCTCCCGTAT	-	[104]
HNE	DNA I DNA, 44 nt	TAGCGATACTGCGTGGGTTGGGGCGGGTAGGGCCAGCAGTCTCG	17 nM	[105]
HGF	H38-15 DNA, 59 nt	GCGCCAGCTTTGCTGATGGGTGGCCACCCTTGCCCTGGGTTTGAATTTCGATCCTATCG	19 nM	[106]
Leptin	Lep3 DNA, 40 nt	GTTAATGGGGGATCTCGCGGCCGTTCTTGTTGCTTATACA	0.3 μM	[107]
Oncostatin M	ADR58 2′-F-RNA, 33 nt	GAA*CC*GG*CCC*AG*C*AGA*CU*G*CU*GA*C*GG*C*A*C*GA*UC*	7 nM	[108]

All modified nucleosides are marked by italics. Bn, 5-(*N*-benzylcarboxamide)-2′-deoxyuridine; Nap, 5-[*N*-(1-naphthylmethyl)carboxamide]-2′-deoxyuridine; Pe, 5-[*N*-(phenyl-2-ethyl)carboxamide]-2′-deoxyuridine; iT, 3′-thymidine residue attached via ‘inverted’ 3′-3′ phosphodiester linkage; 2′-F-RNA, RNA with 2′-fluoro pyrimidine nucleotides; mRfY, RNA with 2′-O-methyl purine and 2′-fluoro pyrimidine nucleotides.

**Table 2:** Aptasensors for detection of protein biomarkers associated with rheumatic disorders.

Target	Sensor Type	Working Range	Samples	Ref.
CRP	SPR	500–1000 ng/mL	Buffer solution	[73]
	Square-wave voltammetry	25–250 pg/mL	10% spiked serum	[110]
	Fluorescent	10 ng/mL–100 μg/mL	1% spiked serum	[111]
	Electrochemical sandwich assay	0.1–50 μg/mL	10% spiked serum	[114]
	Fluorescent sandwich-assay	0.4–10 μg/mL	1% spiked serum	[116]
	Square-wave voltammetry	0.005–125 ng/mL	0.2% clinical and spiked serum	[115]
	non-Faradaic impedance spectroscopy	100–500 pg/mL	Buffer solution	[109]
	Isotachophoresis with fluorescent detection	-	5% spiked serum	[117]
	Luminescent sandwich-assay	0.0125–10 μg/mL	Buffer solution	[75]
	Field-effect-transistor	0.625–10 μg/mL	Buffer solution	[118]
	SPR	0.25 ng/mL–2.5 μg/mL	1% spiked serum	[76]
	Fluorescent	12.5 ng/mL–5 μg/mL	Buffer solution	[119]
	Lossy mode resonance	-	Buffer solution	[120]
TNFα	Differential pulse voltammetry	10 pg/mL–40 μg/mL	10% clinical serum	[121]
	Quantum dots-based photoluminescence	1.7–400 ng/mL	10% spiked serum	[122]
	Aptameric graphene field-effect transistor	-	Buffer solution	[123]
	Alternating current voltammetry	1.75 ng/mL–8.75 μg/mL	Diluted saliva and urine samples	[124]
	Square-wave voltammetry	10–100 ng/mL	Diluted spiked blood	[125]
VEGF	Colorimetric	100–1 × 10^5^ pg/mL	Clinical serum samples	[126]
	Chemiluminescent sandwich assay	1–20 ng/mL	Cell culture medium	[82]
	Colorimetric	0.5–225 pg/mL	12.5% spiked serum	[127]
	Colorimetric	3.7–148 pg/mL	Buffer solution	[128]
	Colorimetric, aptazyme-based	0.1–40 nM	1% spiked serum	[129]
	Chemiluminescent	-	10% spiked serum	[130]
	pH-Meter based	0.8–480 pg/mL	1% serum, centrifuged	[131]
	Glucose meter based	3–100 pg/mL	10% clinical serum	[132]
IL-17RA	Impedimetric	10–10,000 pg/mL	10% spiked serum	[133]
IL-6	Aptameric graphene field-effect transistor	-	Buffer solution	[134]
	Impedimetric	5 pg/mL–100 ng/mL	50% patients’ serum	[135]
	Au-NP aptamer-based sandwich-assay	3.3–125 μg/mL	Buffer solution	[136]
sIL-2Rα	Au-NP colorimetric	25 ng/mL–2.5 μg/mL	10% spiked serum	[137]
IL-8	On-chip rolling cycle amplification	7.5–120 pg/mL	Buffer solution	[138]
DKK1	Aptamer-based ELISA	62.5–4000 pg/mL	10% clinical serum	[95]
CTGF	Aptamer-based biolayer interferometry ELISA	1.1–112 ng/mL	10% spiked serum	[97]
Osteopontin	Lateral flow	10–500 ng/mL	10% spiked serum	[139]
Visfatin	non-Faradaic impedance spectroscopy	1–50 ng/mL	20% filtered spiked serum	[101]
MMP-9	Quartz crystal microbalance	92 pg/mL–230 ng/mL	2–0.25% spiked serum	[103]
CTxI	Fluorescent	-	Buffer solution	[104]
HNE	Fluorescent	1.3 ng/mL–2 μg/mL	Buffer solution	[140]
	Colorimetric	31.2 ng/mL–3.1 μg/mL	Buffer solution	[141]
	Capillary electrophoresis coupled with laser-induced fluorescence	15.6 ng/mL–15.6 μg/mL	1% spiked serum	[142]

## Abbreviations

ALF	Acute liver failure
ALI	Acute lung injury
ANCAs	Anti-neutrophil cytoplasmic antibodies
AS	Ankylosing spondylitis
AuNP	Gold nanoparticles
BLI	Biolayer interferometry
BMD	Bone mineral density
BSA	Bovine serum albumin
CE	Capillary electrophoresis
CEA	Carcinoembryonic antigen
CRP	C-reactive protein
CTGF	Connective tissue growth factor
CTX-I	C-terminal telopeptide
DKK-1	Dikkopf 1
DNMT1	DNA methyltransferase 1
EGFR	Epidermal growth factor receptor
ELISA	Enzyme linked immunosorbent assay
FDA	U.S. Food and Drug Administration
FET	Field effect transistor
FRET	Förster resonance energy transfer
GFET	Graphene-based field effect transistor
GO-SELEX	Graphene oxide-based SELEX
HGF	Hepatocyte growth factor
HNE	Human neutrophil elastase
HSA	Human serum albumin
LOD	Limit of detection
MMP-9	Matrix metalloproteinase 9
NAMPT	Nicotinamide phosphoribosyl transferase
OP	Osteoporosis
OPG	Osteoprotegerin
OPN	Osteopontin
OSM	Oncostatin M
PDGF-BB	Platelet derived growth factor-BB
PEG	Polyethylene glycol
PoC	Point-of-care
QD	Quantum dots
RA	Rheumatoid arthritis
RANK	Receptor activator of nuclear factor kappa-Β
SELEX	Systematic evolution of ligands by exponential enrichment
SERS	Surface-enhanced Raman spectroscopy
SLE	Systemic lupus erythematosus
Sn	Sclerostin
SOMAmer	Slow off-rate modified aptamer
SPR	Surface plasmon resonance
TMB	tetramethylbenzidine
TNFα	Tumor necrosis factor α
VEGF	Vascular endothelial growth factor

## References

[1] Tuerk C., Gold L. (1990). Systematic evolution of ligands by exponential enrichment: RNA ligands to bacteriophage T4 DNA polymerase. Science.

[2] Robertson D.L., Joyce G.F. (1990). Selection in vitro of an RNA enzyme that specifically cleaves single-stranded DNA. Nature.

[3] Ellington A.D., Szostak J.W. (1990). In vitro selection of RNA molecules that bind specific ligands. Nature.

[4] Nimjee S.M., White R.R., Becker R.C., Sullenger B.A. (2017). Aptamers as Therapeutics. Annu. Rev. Pharmacol. Toxicol..

[5] Zhang Y., Lai B., Juhas M. (2019). Recent advances in aptamer discovery and applications. Molecules.

[6] Adachi T., Nakamura Y. (2019). Aptamers: A review of their chemical properties and modifications for therapeutic application. Molecules.

[7] Haßel S.K., Mayer G. (2019). Aptamers as therapeutic agents: Has the initial euphoria subsided?. Mol. Diagnosis Ther..

[8] Kumar Kulabhusan P., Hussain B., Yüce M. (2020). Current perspectives on aptamers as diagnostic tools and therapeutic agents. Pharmaceutics.

[9] Kou X., Zhang X., Shao X., Jiang C., Ning L. (2020). Recent advances in optical aptasensor technology for amplification strategies in cancer diagnostics. Anal. Bioanal. Chem..

[10] Pirzada M., Altintas Z. (2020). Recent progress in optical sensors for biomedical diagnostics. Micromachines.

[11] Yan S.R., Foroughi M.M., Safaei M., Jahani S., Ebrahimpour N., Borhani F., Rezaei Zade Baravati N., Aramesh-Boroujeni Z., Foong L.K. (2020). A review: Recent advances in ultrasensitive and highly specific recognition aptasensors with various detection strategies. Int. J. Biol. Macromol..

[12] Sharma T.K., Bruno J.G., Dhiman A. (2017). ABCs of DNA aptamer and related assay development. Biotechnol. Adv..

[13] Kalra P., Dhiman A., Cho W.C., Bruno J.G., Sharma T.K. (2018). Simple methods and rational design for enhancing aptamer sensitivity and specificity. Front. Mol. Biosci..

[14] Baker M. (2015). Blame it on the antibodies. Nature.

[15] Bradbury A., Plückthun A. (2015). Reproducibility: Standardize antibodies used in research. Nature.

[16] Weller M.G. (2016). Quality issues of research antibodies. Anal. Chem. Insights.

[17] Maimaitiyiming Y., Hong D.F., Yang C., Naranmandura H. (2019). Novel insights into the role of aptamers in the fight against cancer. J. Cancer Res. Clin. Oncol..

[18] Ponce A.T., Hong K.L. (2019). A Mini-Review: Clinical development and potential of aptamers for thrombotic events treatment and monitoring. Biomedicines.

[19] Davydova A., Vorobjeva M., Pyshnyi D., Altman S., Vlassov V., Venyaminova A. (2016). Aptamers against pathogenic microorganisms. Crit. Rev. Microbiol..

[20] Park K.S. (2018). Nucleic acid aptamer-based methods for diagnosis of infections. Biosens. Bioelectron..

[21] Aletaha D., Maa J., Chen S., Park S.-H., Nicholls D., Florentinus S., Furtner D., Smolen J.S. (2019). Effect of disease duration and prior disease-modifying antirheumatic drug use on treatment outcomes in patients with rheumatoid arthritis. Ann. Rheum. Dis..

[22] Deminger A., Klingberg E., Geijer M., Göthlin J., Hedberg M., Rehnberg E., Carlsten H., Jacobsson L.T., Forsblad-d’Elia H. (2018). A five-year prospective study of spinal radiographic progression and its predictors in men and women with ankylosing spondylitis. Arthritis Res. Ther..

[23] Landewé R., Nurminen T., Davies O., Baeten D. (2018). A single determination of C-reactive protein does not suffice to declare a patient with a diagnosis of axial spondyloarthritis “CRP-negative”. Arthritis Res. Ther..

[24] Gravallese E.M., Schett G. (2018). Effects of the IL-23–IL-17 pathway on bone in spondyloarthritis. Nat. Rev. Rheumatol..

[25] Mei Y., Pan F., Gao J., Ge R., Duan Z., Zeng Z., Liao F., Xia G., Wang S., Xu S. (2011). Increased serum IL-17 and IL-23 in the patient with ankylosing spondylitis. Clin. Rheumatol..

[26] Bayat P., Nosrati R., Alibolandi M., Rafatpanah H., Abnous K., Khedri M., Ramezani M. (2018). SELEX methods on the road to protein targeting with nucleic acid aptamers. Biochimie.

[27] Ali M.H., Elsherbiny M.E., Emara M. (2019). Updates on aptamer research. Int. J. Mol. Sci..

[28] Komarova N., Kuznetsov A. (2019). Inside the Black Box: What Makes SELEX Better?. Molecules.

[29] Elskens J.P., Elskens J.M., Madder A. (2020). Chemical Modification of Aptamers for Increased Binding Affinity in Diagnostic Applications: Current Status and Future Prospects. Int. J. Mol. Sci..

[30] Vorobyeva M., Davydova A., Vorobjev P., Pyshnyi D., Venyaminova A. (2018). Key Aspects of Nucleic Acid Library Design for in Vitro Selection. Int. J. Mol. Sci..

[31] Röthlisberger P., Hollenstein M. (2018). Aptamer chemistry. Adv. Drug Deliv. Rev..

[32] Odeh F., Nsairat H., Alshaer W., Ismail M.A., Esawi E., Qaqish B., Bawab A.A., Ismail S.I. (2020). Aptamers chemistry: Chemical modifications and conjugation strategies. Molecules.

[33] Moreno A., Pitoc G.A., Ganson N.J., Layzer J.M., Hershfield M.S., Tarantal A.F., Sullenger B.A. (2019). Anti-PEG Antibodies inhibit the anticoagulant activity of PEGylated aptamers. Cell Chem. Biol..

[34] Zhang P., Sun F., Liu S., Jiang S. (2016). Anti-PEG antibodies in the clinic: Current issues and beyond PEGylation. J. Control. Release.

[35] Vasilescu A., Marty J.L. (2016). Electrochemical aptasensors for the assessment of food quality and safety. Trends Anal. Chem..

[36] Schmitz F.R.W., Valério A., de Oliveira D., Hotza D. (2020). An overview and future prospects on aptamers for food safety. Appl. Microbiol. Biotechnol..

[37] Mishra G., Sharma V., Mishra R. (2018). Electrochemical aptasensors for food and environmental safeguarding: A review. Biosensors.

[38] Li Z., Mohamed M.A., Vinu Mohan A.M., Zhu Z., Sharma V., Mishra G.K., Mishra R.K. (2019). Application of electrochemical aptasensors toward clinical diagnostics, food, and environmental monitoring: Review. Sensors.

[39] McConnell E.M., Nguyen J., Li Y. (2020). Aptamer-based biosensors for environmental monitoring. Front. Chem..

[40] Heydari M., Gholoobi A., Ranjbar G., Rahbar N., Sany S.B.T., Mobarhan M.G., Ferns G.A., Rezayi M. (2020). Aptamers as potential recognition elements for detection of vitamins and minerals: A systematic and critical review. Crit. Rev. Clin. Lab. Sci..

[41] Şahin S., Caglayan M.O., Üstündağ Z. (2020). Recent advances in aptamer-based sensors for breast cancer diagnosis: Special cases for nanomaterial-based VEGF, HER2, and MUC1 aptasensors. Microchim. Acta.

[42] Han K., Liu T., Wang Y., Miao P. (2016). Electrochemical aptasensors for detection of small molecules, macromolecules, and cells. Rev. Anal. Chem..

[43] Xu Y., Cheng G., He P., Fang Y. (2009). A Review: Electrochemical aptasensors with various detection strategies. Electroanalysis.

[44] Reid R., Chatterjee B., Das S.J., Ghosh S., Sharma T.K. (2020). Application of aptamers as molecular recognition elements in lateral flow assays. Anal. Biochem..

[45] Citartan M., Tang T.-H. (2019). Recent developments of aptasensors expedient for point-of-care (POC) diagnostics. Talanta.

[46] Liu J.J., Li R., Gan Y.Z., Zhang R.J., Li J., Cai Y.M., Zhao J.X., Liao H., Xu J., Shi L.J. (2019). Clinical deep remission and related factors in a large cohort of patients with rheumatoid arthritis. Chin. Med. J..

[47] Chung H.Y., Chui E.T.F., Lee K.H., Tsang H.H.L., Chan S.C.W., Lau C.S. (2019). ASDAS is associated with both the extent and intensity of DW-MRI spinal inflammation in active axial spondyloarthritis. RMD Open.

[48] Yahagi A., Saika T., Hirano H., Takai-Imamura M., Tsuji F., Aono H., Iseki M., Morita Y., Igarashi H., Saeki Y. (2019). IL-6-PAD4 axis in the earliest phase of arthritis in knock-in gp130F759 mice, a model for rheumatoid arthritis. RMD Open.

[49] Tanaka Y., Sugiyama N., Toyoizumi S., Lukic T., Lamba M., Zhang R., Chen C., Stock T., Valdez H., Mojcik C. (2019). Modified-versus immediate-release tofacitinib in Japanese rheumatoid arthritis patients: A randomized, phase III, non-inferiority study. Rheumatology.

[50] Su J., Cui L., Yang W., Shi H., Jin C., Shu R., Li H., Zeng X., Wu S., Gao X. (2019). Baseline high-sensitivity C-reactive protein predicts the risk of incident ankylosing spondylitis: Results of a community-based prospective study. PLoS ONE.

[51] Chan F.L.Y., Lester S., Whittle S.L., Hill C.L. (2019). The utility of ESR, CRP and platelets in the diagnosis of GCA. BMC Rheumatol..

[52] Ing E.B., Lahaie Luna G., Toren A., Ing R., Chen J., Arora N., Torun N., Jakpor O.A., Fraser J.A., Tyndel F.J. (2017). Multivariate prediction model for suspected giant cell arteritis: Development and validation. Clin. Ophthalmol..

[53] Becker M., Graf N., Sauter R., Allanore Y., Curram J., Denton C.P., Khanna D., Matucci-Cerinic M., de Oliveira Pena J., Pope J.E. (2019). Predictors of disease worsening defined by progression of organ damage in diffuse systemic sclerosis: A European Scleroderma Trials and Research (EUSTAR) analysis. Ann. Rheum. Dis..

[54] Lis-Święty A., Widuchowska M., Brzezińska-Wcisło L., Kucharz E. (2018). High acute phase protein levels correlate with pulmonary and skin involvement in patients with diffuse systemic sclerosis. J. Int. Med. Res..

[55] Wang J., Niu R., Jiang L., Wang Y., Shao X., Wu M., Ma Y. (2019). The diagnostic values of C-reactive protein and procalcitonin in identifying systemic lupus erythematosus infection and disease activity. Medicine.

[56] Littlejohn E., Marder W., Lewis E., Francis S., Jackish J., McCune W.J., Somers E.C. (2018). The ratio of erythrocyte sedimentation rate to C-reactive protein is useful in distinguishing infection from flare in systemic lupus erythematosus patients presenting with fever. Lupus.

[57] Bay-Jensen A.C., Platt A., Jenkins M.A., Weinblatt M.E., Byrjalsen I., Musa K., Genovese M.C., Karsdal M.A. (2019). Tissue metabolite of type I collagen, C1M, and CRP predicts structural progression of rheumatoid arthritis. BMC Rheumatol..

[58] Yeh J.-C., Wu C.-C., Choy C.-S., Chang S.-W., Liou J.-C., Chen K.-S., Tung T.-H., Lin W.-N., Hsieh C.-Y., Ho C.-T. (2018). Non-hepatic alkaline phosphatase, hs-CRP and progression of vertebral fracture in patients with rheumatoid arthritis: A population-based longitudinal study. J. Clin. Med..

[59] Yu Z., Kim S.C., Vanni K., Huang J., Desai R., Murphy S.N., Solomon D.H., Liao K.P. (2018). Association between inflammation and systolic blood pressure in RA compared to patients without RA. Arthritis Res. Ther..

[60] Azevedo S., Santos-Faria D., Leite Silva J., Ramos Rodrigues J., Sousa Neves J., Peixoto D., Tavares-Costa J., Alcino S., Afonso C., Teixeira F. (2019). Obesity, metabolic syndrome and other comorbidities in rheumatoid arthritis and psoriatic arthritis: Influence on disease activity and quality of life. Acta Reumatol. Port..

[61] Ferguson L.D., Siebert S., McInnes I.B., Sattar N. (2019). Cardiometabolic comorbidities in RA and PsA: Lessons learned and future directions. Nat. Rev. Rheumatol..

[62] Dimitroulas T., Hodson J., Sandoo A., Smith J., Kitas G.D. (2017). Endothelial injury in rheumatoid arthritis: A crosstalk between dimethylarginines and systemic inflammation. Arthritis Res. Ther..

[63] Pan L., Wang T. (2017). Features of cardiac remodeling in patients with acute coronary syndrome complicated with rheumatoid arthritis. Sci. Rep..

[64] Nash P., Ohson K., Walsh J., Delev N., Nguyen D., Teng L., Gómez-Reino J.J., Aelion J.A. (2018). Early and sustained efficacy with apremilast monotherapy in biological-naïve patients with psoriatic arthritis: A phase IIIB, randomised controlled trial (ACTIVE). Ann. Rheum. Dis..

[65] McInnes I.B., Chakravarty S.D., Apaolaza I., Kafka S., Hsia E.C., You Y., Kavanaugh A. (2019). Efficacy of ustekinumab in biologic-naïve patients with psoriatic arthritis by prior treatment exposure and disease duration: Data from PSUMMIT 1 and PSUMMIT 2. RMD Open.

[66] Park J.W., Kim H.-A., Shin K., Park Y.-B., Kim T.-H., Song Y.W., Lee E.Y. (2019). Effects of tapering tumor necrosis factor inhibitor on the achievement of inactive disease in patients with axial spondyloarthritis: A nationwide cohort study. Arthritis Res. Ther..

[67] Cohen S., Pablos J.L., Pavelka K., Müller G.A., Matsumoto A., Kivitz A., Wang H., Krishnan E. (2019). An open-label extension study to demonstrate long-term safety and efficacy of ABP 501 in patients with rheumatoid arthritis. Arthritis Res. Ther..

[68] Park W., Božić-Majstorović L., Milakovic D., Berrocal Kasay A., El-Khouri E.C., Irazoque-Palazuelos F., Molina F.F.C., Shesternya P., Miranda P., Medina-Rodriguez F.G. (2018). Comparison of biosimilar CT-P10 and innovator rituximab in patients with rheumatoid arthritis: A randomized controlled Phase 3 trial. MAbs.

[69] Fleischmann R.M., Alten R., Pileckyte M., Lobello K., Hua S.Y., Cronenberger C., Alvarez D., Bock A.E., Sewell K.L. (2018). A comparative clinical study of PF-06410293, a candidate adalimumab biosimilar, and adalimumab reference product (Humira^®^) in the treatment of active rheumatoid arthritis. Arthritis Res. Ther..

[70] Taylor P.C., Saurigny D., Vencovsky J., Takeuchi T., Nakamura T., Matsievskaia G., Hunt B., Wagner T., Souberbielle B. (2019). Efficacy and safety of namilumab, a human monoclonal antibody against granulocyte-macrophage colony-stimulating factor (GM-CSF) ligand in patients with rheumatoid arthritis (RA) with either an inadequate response to background methotrexate therapy or an i. Arthritis Res. Ther..

[71] Braun J., Deodhar A., Landewé R., Baraliakos X., Miceli-Richard C., Sieper J., Quebe-Fehling E., Martin R., Porter B., Gandhi K.K. (2018). Impact of baseline C-reactive protein levels on the response to secukinumab in ankylosing spondylitis: 3-year pooled data from two phase III studies. RMD Open.

[72] Strand V., Alemao E., Lehman T., Johnsen A., Banerjee S., Ahmad H.A., Mease P.J. (2018). Improved patient-reported outcomes in patients with psoriatic arthritis treated with abatacept: Results from a phase 3 trial. Arthritis Res. Ther..

[73] Bini A., Centi S., Tombelli S., Minunni M., Mascini M. (2008). Development of an optical RNA-based aptasensor for C-reactive protein. Anal. Bioanal. Chem..

[74] Orito N., Umekage S., Sato K., Kawauchi S., Tanaka H., Sakai E., Tanaka T., Kikuchi Y. (2012). High-affinity RNA aptamers to C-reactive protein (CRP): Newly developed pre-elution methods for aptamer selection. J. Phys. Conf. Ser..

[75] Huang C., Lin H., Shiesh S., Lee G. (2010). Integrated microfluidic system for rapid screening of CRP aptamers utilizing systematic evolution of ligands by exponential enrichment (SELEX). Biosens. Bioelectron..

[76] Wu B., Jiang R., Wang Q., Huang J., Yang X., Wang K., Li W., Chen N., Li Q. (2016). Detection of C-reactive protein using nanoparticle-enhanced surface plasmon resonance using an aptamer-antibody sandwich assay. Chem. Commun..

[77] Yang X., Wang Y., Wang K., Wang Q., Wang P., Lin M., Chen N., Tan Y. (2014). DNA aptamer-based surface plasmon resonance sensing of human C-reactive protein. RSC Adv..

[78] Lai W.Y., Wang J.W., Huang B.T., Lin E.P.Y., Yang P.C. (2019). A Novel TNF-α-targeting aptamer for TNF-α-mediated acute lung injury and acute liver failure. Theranostics.

[79] Orava E.W., Jarvik N., Shek Y.L., Sidhu S.S., Gariépy J. (2013). A Short DNA aptamer that recognizes TNFα and blocks its activity in vitro. ACS Chem. Biol..

[80] Yan X., Gao X., Zhang Z. (2004). Isolation and characterization of 2′-amino-modified RNA aptamers for human TNFalpha. Genom. Proteom. Bioinform..

[81] Mashayekhi K., Ganji A., Sankian M. (2020). Designing a new dimerized anti human TNF-α aptamer with blocking activity. Biotechnol. Prog..

[82] Shan S., He Z., Mao S., Jie M., Yi L., Lin J.M. (2017). Quantitative determination of VEGF165 in cell culture medium by aptamer sandwich based chemiluminescence assay. Talanta.

[83] Mori T., Oguro A., Ohtsu T., Nakamura Y. (2004). RNA aptamers selected against the receptor activator of NF-κB acquire general affinity to proteins of the tumor necrosis factor receptor family. Nucleic Acids Res..

[84] Chen L., Li D.Q., Zhong J., Wu X.L., Chen Q., Peng H., Liu S.Q. (2011). IL-17RA aptamer-mediated repression of IL-6 inhibits synovium inflammation in a murine model of osteoarthritis. Osteoarthr. Cartil..

[85] Ishiguro A., Akiyama T., Adachi H., Inoue J.I., Nakamura Y. (2011). Therapeutic potential of anti-interleukin-17A aptamer: Suppression of interleukin-17A signaling and attenuation of autoimmunity in two mouse models. Arthritis Rheum..

[86] Adachi H., Ishiguro A., Hamada M., Sakota E., Asai K., Nakamura Y. (2011). Antagonistic RNA aptamer specific to a heterodimeric form of human interleukin-17A/F. Biochimie.

[87] Gupta S., Hirota M., Waugh S.M., Murakami I., Suzuki T., Muraguchi M., Shibamori M., Ishikawa Y., Jarvis T.C., Carter J.D. (2014). Chemically modified DNA aptamers bind interleukin-6 with high affinity and inhibit signaling by blocking its interaction with interleukin-6 receptor. J. Biol. Chem..

[88] Meyer C., Eydeler-Haeder K., Magbanua E., Tijana Z., Piganeau N., Lorenzen I., Grötzinger J., Mayer G., Rose-John S., Hahn U. (2012). Interleukin-6 receptor specific RNA aptamers for cargo delivery into target cells. RNA Biol..

[89] Meyer C., Berg K., Eydeler-Haeder K., Lorenzen I., Grötzinger J., Rose-John S., Hahn U. (2014). Stabilized interleukin-6 receptor binding RNA aptamers. RNA Biol..

[90] Mittelberger F., Meyer C., Waetzig G.H., Zacharias M., Valentini E., Svergun D.I., Rose-john S., Hahn U. (2015). RAID3—An interleukin-6 receptor-binding aptamer with post-selective modification-resistant affinity. RNA Biol..

[91] Lenn J.D., Neil J., Donahue C., Demock K., Tibbetts C.V., Cote-Sierra J., Smith S.H., Rubenstein D., Therrien J.P., Pendergrast P.S. (2018). RNA aptamer delivery through intact human skin. J. Invest. Dermatol..

[92] Kim S., Kim J.H., Yoon S., Kim K.S., Yoon M.Y., Yoon D.Y., Kim D.E. (2010). Generation of antagonistic RNA aptamers specific to proinflammatory cytokine interleukin-32. Bull. Korean Chem. Soc..

[93] Sung H.J., Choi S., Lee J.W., Ok C.Y., Bae Y.S., Kim Y.H., Lee W., Heo K., Kim I.H. (2014). Inhibition of human neutrophil activity by an RNA aptamer bound to interleukin-8. Biomaterials.

[94] Ren X., Gelinas A.D., von Carlowitz I., Janjic N., Pyle A.M. (2017). Structural basis for IL-1α recognition by a modified DNA aptamer that specifically inhibits IL-1α signaling. Nat. Commun..

[95] Zhou Y., Li W., Tseng Y., Zhang J., Liu J. (2019). Developing slow-off dickkopf-1 aptamers for early-diagnosis of hepatocellular carcinoma. Talanta.

[96] Shum K.T., Chan C., Leung C.M., Tanner J.A. (2011). Identification of a DNA aptamer that inhibits sclerostin’s antagonistic effect on Wnt signalling. Biochem. J..

[97] Gao S., Hu W., Zheng X., Cai S., Wu J. (2019). Functionalized aptamer with an antiparallel G-quadruplex: Structural remodeling, recognition mechanism, and diagnostic applications targeting CTGF. Biosens. Bioelectron..

[98] Li S., Huo Y., Tian H., Zhang Q., Lv Y., Hao Z. (2015). In vitro selection and characterization of deoxyribonucleic acid aptamers against connective tissue growth factor. Biochem. Biophys. Res. Commun..

[99] Mi Z., Guo H., Russell M.B., Liu Y., Sullenger B.A., Kuo P.C. (2009). RNA aptamer blockade of osteopontin inhibits growth and metastasis of MDA-MB231 breast cancer cells. Mol. Ther..

[100] Mor-Vaknin N., Saha A., Legendre M., Carmona-Rivera C., Amin M.A., Rabquer B.J., Gonzales-Hernandez M.J., Jorns J., Mohan S., Yalavarthi S. (2017). DEK-targeting DNA aptamers as therapeutics for inflammatory arthritis. Nat. Commun..

[101] Park J.-W., Saravan Kallempudi S., Niazi J.H., Gurbuz Y., Youn B.-S., Gu M.B. (2012). Rapid and sensitive detection of Nampt (PBEF/visfatin) in human serum using an ssDNA aptamer-based capacitive biosensor. Biosens. Bioelectron..

[102] Da Rocha Gomes S., Miguel J., Azéma L., Eimer S., Ries C., Dausse E., Loiseau H., Allard M., Toulmé J.J. (2012). 99mTc-MAG3-aptamer for imaging human tumors associated with high level of matrix metalloprotease-9. Bioconjug. Chem..

[103] Scarano S., Dausse E., Crispo F., Toulmé J.-J., Minunni M. (2015). Design of a dual aptamer-based recognition strategy for human matrix metalloproteinase 9 protein by piezoelectric biosensors. Anal. Chim. Acta.

[104] Bruno J.G., Carrillo M.P., Phillips T., Hanson D., Bohmann J.A. (2011). DNA aptamer beacon assay for C-telopeptide and handheld fluorometer to monitor bone resorption. J. Fluoresc..

[105] Lin Y., Padmapriya A., Morden K.M., Jayasena S.D. (1995). Peptide conjugation to an in vitro-selected DNA ligand improves enzyme inhibition. Proc. Natl. Acad. Sci. USA.

[106] Saito T., Tomida M. (2005). Generation of inhibitory DNA aptamers against human hepatocyte growth factor. DNA Cell Biol..

[107] Ashley J., Li S.F.Y. (2013). Three-dimensional selection of leptin aptamers using capillary electrophoresis and implications for clone validation. Anal. Biochem..

[108] Rhodes A., Deakin A., Spaull J., Coomber B., Aitken A., Life P., Rees S. (2000). The generation and characterization of antagonist RNA aptamers to human oncostatin M. J. Biol. Chem..

[109] Qureshi A., Gurbuz Y., Kallempudi S., Niazi J.H. (2010). Label-free RNA aptamer-based capacitive biosensor for the detection of C-reactive protein. Phys. Chem. Chem. Phys..

[110] Jarczewska M., Rębiś J., Górski Ł., Malinowska E. (2018). Development of DNA aptamer-based sensor for electrochemical detection of C-reactive protein. Talanta.

[111] Pultar J., Sauer U., Domnanich P., Preininger C. (2009). Aptamer-antibody on-chip sandwich immunoassay for detection of CRP in spiked serum. Biosens. Bioelectron..

[112] António M., Ferreira R., Vitorino R., Daniel-da-Silva A.L. (2020). A simple aptamer-based colorimetric assay for rapid detection of C-reactive protein using gold nanoparticles. Talanta.

[113] Xie J., Tang M.Q., Chen J., Zhu Y.H., Lei C.B., He H.W., Xu X.H. (2020). A sandwich ELISA-like detection of C-reactive protein in blood by citicoline-bovine serum albumin conjugate and aptamer-functionalized gold nanoparticles nanozyme. Talanta.

[114] Centi S., Sanmartin L.B., Tombelli S., Palchetti I., Mascini M. (2009). Detection of C reactive protein (CRP) in serum by an electrochemical aptamer-based sandwich assay. Electroanalysis.

[115] Wang J., Guo J., Zhang J., Zhang W., Zhang Y. (2017). RNA aptamer-based electrochemical aptasensor for C-reactive protein detection using functionalized silica microspheres as immunoprobes. Biosens. Bioelectron..

[116] Bernard E.D., Nguyen K.C., DeRosa M.C., Tayabali A.F., Aranda-Rodriguez R. (2015). Development of a bead-based aptamer/antibody detection system for C-reactive protein. Anal. Biochem..

[117] Eid C., Palko J.W., Katilius E., Santiago J.G. (2015). Rapid Slow Off-Rate Modified Aptamer (SOMAmer)-Based Detection of C-Reactive Protein Using Isotachophoresis and an Ionic Spacer. Anal. Chem..

[118] Kao W.-C., Chen Y.-W., Chu C.-H., Chang W.-H., Shiesh S.-C., Wang Y.-L., Lee G.-B. (2017). Detection of C-reactive protein on an integrated microfluidic system by utilizing field-effect transistors and aptamers. Biomicrofluidics.

[119] Wu B., Chen N., Wang Q., Yang X., Wang K., Li W., Li Q., Liu W., Fang H. (2016). A simple label-free aptamer-based method for C-reactive protein detection. Anal. Methods.

[120] Zubiate P., Zamarreño C.R., Sánchez P., Matias I.R., Arregui F.J. (2017). High sensitive and selective C-reactive protein detection by means of lossy mode resonance based optical fiber devices. Biosens. Bioelectron..

[121] Ghalehno M.H., Mirzaei M., Torkzadeh-Mahani M. (2018). Aptamer-based determination of tumor necrosis factor α using a screen-printed graphite electrode modified with gold hexacyanoferrate. Mikrochim. Acta.

[122] Ghosh S., Datta D., Chaudhry S., Dutta M., Stroscio M.A. (2018). Rapid detection of tumor necrosis factor-alpha using quantum dot-based optical aptasensor. IEEE Trans. Nanobiosci..

[123] Hao Z., Wang Z., Li Y., Zhu Y., Wang X., De Moraes C.G., Pan Y., Zhao X., Lin Q. (2018). Measurement of cytokine biomarkers using an aptamer-based affinity graphene nanosensor on a flexible substrate toward wearable applications. Nanoscale.

[124] Mayer M.D., Lai R.Y. (2018). Effects of redox label location on the performance of an electrochemical aptamer-based tumor necrosis factor-alpha sensor. Talanta.

[125] Liu Y., Zhou Q., Revzin A. (2013). An aptasensor for electrochemical detection of tumor necrosis factor in human blood. Analyst.

[126] Dong J., He L., Wang Y., Yu F., Yu S., Liu L., Wang J., Tian Y., Qu L., Han R. (2020). A highly sensitive colorimetric aptasensor for the detection of the vascular endothelial growth factor in human serum. Spectrochim. Acta Part A Mol. Biomol. Spectrosc..

[127] Zhang H., Peng L., Li M., Ma J., Qi S., Chen H., Zhou L., Chen X. (2017). A label-free colorimetric biosensor for sensitive detection of vascular endothelial growth factor-165. Analyst.

[128] Chang C.C., Chen C.Y., Chuang T.L., Wu T.H., Wei S.C., Liao H., Lin C.W. (2016). Aptamer-based colorimetric detection of proteins using a branched DNA cascade amplification strategy and unmodified gold nanoparticles. Biosens. Bioelectron..

[129] Wu D., Gao T., Lei L., Yang D., Mao X., Li G. (2016). Colorimetric detection of proteins based on target-induced activation of aptazyme. Anal. Chim. Acta.

[130] Freeman R., Girsh J., Fang-Ju Jou A., Ho J.A.A., Hug T., Dernedde J., Willner I. (2012). Optical aptasensors for the analysis of the vascular endothelial growth factor (VEGF). Anal. Chem..

[131] Xu H., Kou F., Ye H., Wang Z., Huang S., Liu X., Zhu X., Lin Z., Chen G. (2017). Highly sensitive antibody-aptamer sensor for vascular endothelial growth factor based on hybridization chain reaction and pH meter/indicator. Talanta.

[132] Zhu X., Kou F., Xu H., Lin L., Yang G., Lin Z. (2017). A highly sensitive aptamer-immunoassay for vascular endothelial growth factor coupled with portable glucose meter and hybridization chain reaction. Sens. Actuators B Chem..

[133] Jo H., Kim S.H.S.K., Youn H., Lee H., Lee K., Jeong J., Mok J., Kim S.H.S.K., Park H.S., Ban C. (2016). A highly sensitive and selective impedimetric aptasensor for interleukin-17 receptor A. Biosens. Bioelectron..

[134] Hao Z., Pan Y., Huang C., Wang Z., Zhao X. (2019). Sensitive detection of lung cancer biomarkers using an aptameric graphene-based nanosensor with enhanced stability. Biomed. Microdev..

[135] Tertis M., Leva P.I., Bogdan D., Suciu M., Graur F., Cristea C. (2019). Impedimetric aptasensor for the label-free and selective detection of Interleukin-6 for colorectal cancer screening. Biosens. Bioelectron..

[136] Giorgi-Coll S., Marín M.J., Sule O., Hutchinson P.J., Carpenter K.L.H. (2020). Aptamer-modified gold nanoparticles for rapid aggregation-based detection of inflammation: An optical assay for interleukin-6. Microchim. Acta.

[137] Jeon J., Jo H., Her J., Youn H., Park J., Jo J., Lee J., Chang C.L., Ban C. (2019). A Rapid colorimetric sensor for soluble Interleukin-2 receptor α, based on aptamer-adsorbed AuNP. ChemBioChem.

[138] Zhang W., He Z., Yi L., Mao S., Li H., Lin J.M. (2018). A dual-functional microfluidic chip for on-line detection of interleukin-8 based on rolling circle amplification. Biosens. Bioelectron..

[139] Mukama O., Wu W., Wu J., Lu X., Liu Y., Liu Y., Liu J., Zeng L. (2020). A highly sensitive and specific lateral flow aptasensor for the detection of human osteopontin. Talanta.

[140] He J.L., Wu Z.S., Zhang S.B., Shen G.L., Yu R.Q. (2010). Fluorescence aptasensor based on competitive-binding for human neutrophil elastase detection. Talanta.

[141] Cheng L., Zhao Q. (2013). Aptamer-capture based assays for human neutrophil elastase. Talanta.

[142] Bai Y., Wang H., Zhao Q. (2017). Detection of human neutrophil elastase by aptamer affinity capillary electrophoresis coupled with laser-induced fluorescence using specified site fluorescently labeled aptamer. Anal. Bioanal. Chem..

[143] Camussi G., Albano E., Tetta C., Bussolino F. (1991). The molecular action of tumor necrosis factor-alpha. Eur. J. Biochem..

[144] Acar L., Atalan N., Karagedik E.H., Ergen A. (2018). Tumour necrosis factor-alpha and nuclear factor-kappa b gene variants in sepsis. Balkan Med. J..

[145] Horiuchi T., Mitoma H., Harashima S. (2010). -i.; Tsukamoto, H.; Shimoda, T. Transmembrane TNF-: Structure, function and interaction with anti-TNF agents. Rheumatology.

[146] Keffer J., Probert L., Cazlaris H., Georgopoulos S., Kaslaris E., Kioussis D., Kollias G. (1991). Transgenic mice expressing human tumour necrosis factor: A predictive genetic model of arthritis. EMBO J..

[147] Zwerina J., Redlich K., Polzer K., Joosten L., Kronke G., Distler J., Hess A., Pundt N., Pap T., Hoffmann O. (2007). TNF-induced structural joint damage is mediated by IL-1. Proc. Natl. Acad. Sci. USA.

[148] Gorth D.J., Shapiro I.M., Risbud M.V. (2019). Transgenic mice overexpressing human TNF-α experience early onset spontaneous intervertebral disc herniation in the absence of overt degeneration. Cell Death Dis..

[149] Merola J.F., Espinoza L.R., Fleischmann R. (2018). Distinguishing rheumatoid arthritis from psoriatic arthritis. RMD Open.

[150] Jung M.K., Lee J.S., Kwak J.E., Shin E.C. (2019). Tumor necrosis factor and regulatory T cells. Yonsei Med. J..

[151] Liu Y., Kwa T., Revzin A. (2012). Simultaneous detection of cell-secreted TNF-α and IFN-γ using micropatterned aptamer-modified electrodes. Biomaterials.

[152] Russo Krauss I., Merlino A., Randazzo A., Novellino E., Mazzarella L., Sica F. (2012). High-resolution structures of two complexes between thrombin and thrombin-binding aptamer shed light on the role of cations in the aptamer inhibitory activity. Nucleic Acids Res..

[153] Dolot R., Lam C.H., Sierant M., Zhao Q., Liu F.-W., Nawrot B., Egli M., Yang X. (2018). Crystal structures of thrombin in complex with chemically modified thrombin DNA aptamers reveal the origins of enhanced affinity. Nucleic Acids Res..

[154] Lee Y.H., Bae S.C. (2018). Correlation between circulating VEGF levels and disease activity in rheumatoid arthritis: A meta-analysis. Z. Rheumatol..

[155] Fromm S., Cunningham C.C., Dunne M.R., Veale D.J., Fearon U., Wade S.M. (2019). Enhanced angiogenic function in response to fibroblasts from psoriatic arthritis synovium compared to rheumatoid arthritis. Arthritis Res. Ther..

[156] Supuran C.T. (2019). Agents for the prevention and treatment of age-related macular degeneration and macular edema: A literature and patent review. Expert Opin. Ther. Pat..

[157] Dehghani S., Nosrati R., Yousefi M., Nezami A., Soltani F., Taghdisi S.M., Abnous K., Alibolandi M., Ramezani M. (2018). Aptamer-based biosensors and nanosensors for the detection of vascular endothelial growth factor (VEGF): A review. Biosens. Bioelectron..

[158] Tanaka Y., Ohira T. (2018). Mechanisms and therapeutic targets for bone damage in rheumatoid arthritis, in particular the RANK-RANKL system. Curr. Opin. Pharmacol..

[159] Yang H., Liu W., Zhou X., Rui H., Zhang H., Liu R. (2019). The association between RANK, RANKL and OPG gene polymorphisms and the risk of rheumatoid arthritis: A case-controlled study and meta-analysis. Biosci. Rep..

[160] Takayanagi H., Ogasawara K., Hida S., Chiba T., Murata S., Sato K., Takaoka A., Yokochi T., Oda H., Tanaka K. (2000). T-cell-mediated regulation of osteoclastogenesis by signalling cross-talk between RANKL and IFN-γ. Nature.

[161] Komatsu N., Okamoto K., Sawa S., Nakashima T., Oh-hora M., Kodama T., Tanaka S., Bluestone J.A., Takayanagi H. (2014). Pathogenic conversion of Foxp3+ T cells into TH17 cells in autoimmune arthritis. Nat. Med..

[162] Itonaga I., Fujikawa Y., Sabokbar A., Murray D.W., Athanasou N.A. (2000). Rheumatoid arthritis synovial macrophage-osteoclast differentiation is osteoprotegerin ligand-dependent. J. Pathol..

[163] Danks L. (2002). Synovial macrophage-osteoclast differentiation in inflammatory arthritis. Ann. Rheum. Dis..

[164] Cummings S.R., Martin J.S., McClung M.R., Siris E.S., Eastell R., Reid I.R., Delmas P., Zoog H.B., Austin M., Wang A. (2009). Denosumab for prevention of fractures in postmenopausal women with osteoporosis. N. Engl. J. Med..

[165] Henry D.H., Costa L., Goldwasser F., Hirsh V., Hungria V., Prausova J., Scagliotti G.V., Sleeboom H., Spencer A., Vadhan-Raj S. (2011). Randomized, double-blind study of denosumab versus zoledronic acid in the treatment of bone metastases in patients with advanced cancer (excluding breast and prostate cancer) or multiple myeloma. J. Clin. Oncol..

[166] LIU W., ZHANG X. (2015). Receptor activator of nuclear factor-κB ligand (RANKL)/RANK/osteoprotegerin system in bone and other tissues (Review). Mol. Med. Rep..

[167] van Hamburg J.P., Tas S.W. (2018). Molecular mechanisms underpinning T helper 17 cell heterogeneity and functions in rheumatoid arthritis. J. Autoimmun..

[168] DeLay M.L., Turner M.J., Klenk E.I., Smith J.A., Sowders D.P., Colbert R.A. (2009). HLA-B27 misfolding and the unfolded protein response augment interleukin-23 production and are associated with Th17 activation in transgenic rats. Arthritis Rheum..

[169] Kenna T.J., Davidson S.I., Duan R., Bradbury L.A., McFarlane J., Smith M., Weedon H., Street S., Thomas R., Thomas G.P. (2012). Enrichment of circulating interleukin-17-secreting interleukin-23 receptor-positive γ/δ T cells in patients with active ankylosing spondylitis. Arthritis Rheum..

[170] Sato K., Suematsu A., Okamoto K., Yamaguchi A., Morishita Y., Kadono Y., Tanaka S., Kodama T., Akira S., Iwakura Y. (2006). Th17 functions as an osteoclastogenic helper T cell subset that links T cell activation and bone destruction. J. Exp. Med..

[171] Schett G., Lories R.J., D’Agostino M.-A., Elewaut D., Kirkham B., Soriano E.R., McGonagle D. (2017). Enthesitis: From pathophysiology to treatment. Nat. Rev. Rheumatol..

[172] Appel H., Maier R., Wu P., Scheer R., Hempfing A., Kayser R., Thiel A., Radbruch A., Loddenkemper C., Sieper J. (2011). Analysis of IL-17+ cells in facet joints of patients with spondyloarthritis suggests that the innate immune pathway might be of greater relevance than the Th17-mediated adaptive immune response. Arthritis Res. Ther..

[173] Braun J., Baraliakos X., Deodhar A., Baeten D., Sieper J., Emery P., Readie A., Martin R., Mpofu S., Richards H.B. (2017). Effect of secukinumab on clinical and radiographic outcomes in ankylosing spondylitis: 2-year results from the randomised phase III MEASURE 1 study. Ann. Rheum. Dis..

[174] Choy E. (2012). Understanding the dynamics: Pathways involved in the pathogenesis of rheumatoid arthritis. Rheumatology.

[175] Heinrich P.C., Behrmann I., Haan S., Hermanns H.M., Müller-Newen G., Schaper F. (2003). Principles of interleukin (IL)-6-type cytokine signalling and its regulation. Biochem. J..

[176] Scheller J., Chalaris A., Schmidt-Arras D., Rose-John S. (2011). The pro- and anti-inflammatory properties of the cytokine interleukin-6. Biochim. Biophys. Acta Mol. Cell Res..

[177] Taga T., Kishimoto T. (1997). gp130 and the interleukin-6 family of cytokines. Annu. Rev. Immunol..

[178] Jostock T., Müllberg J., Özbek S., Atreya R., Blinn G., Voltz N., Fischer M., Neurath M.F., Rose-John S. (2001). Soluble gp130 is the natural inhibitor of soluble interleukin-6 receptor transsignaling responses. Eur. J. Biochem..

[179] Kishimoto T. (1989). The biology of interleukin-6. Blood.

[180] Heinrich P.C., Castell J.V., Andus T. (1990). Interleukin-6 and the acute phase response. Biochem. J..

[181] Wu Q., Zhou X., Huang D., JI Y., Kang F. (2017). IL-6 Enhances osteocyte-mediated osteoclastogenesis by promoting JAK2 and RANKL activity in vitro. Cell. Physiol. Biochem..

[182] McGregor N.E., Murat M., Elango J., Poulton I.J., Walker E.C., Crimeen-Irwin B., Ho P.W.M., Gooi J.H., Martin T.J., Sims N.A. (2019). IL-6 exhibits both cis- and trans-signaling in osteocytes and osteoblasts, but only trans -signaling promotes bone formation and osteoclastogenesis. J. Biol. Chem..

[183] Karateev D.E., Luchikhina E.L. (2019). New possibilities of drug therapy for rheumatoid arthritis: Focus at sarilumab. Alm. Clin. Med..

[184] Hirota M., Murakami I., Ishikawa Y., Suzuki T., Sumida S.I., Ibaragi S., Kasai H., Horai N., Drolet D.W., Gupta S. (2016). Chemically modified interleukin-6 aptamer inhibits development of collagen-induced arthritis in cynomolgus monkeys. Nucleic Acid Ther..

[185] Kruspe S., Meyer C., Hahn U. (2014). Chlorin e6 conjugated Interleukin-6 receptor aptamers selectively kill target cells upon irradiation. Mol. Ther. Nucleic Acids.

[186] Prisner L., Bohn N., Hahn U., Mews A. (2017). Size dependent targeted delivery of gold nanoparticles modified with the IL-6R-specific aptamer AIR-3A to IL-6R-carrying cells. Nanoscale.

[187] Hahn U. (2017). Charomers—Interleukin-6 receptor specific aptamers for cellular internalization and targeted drug delivery. Int. J. Mol. Sci..

[188] Novikov A.A., Aleksandrova E.N., Diatroptova M.A., Nasonov E.L. (2010). Role of cytokines in the pathogenesis of rheumatoid arthritis. Rheumatol. Sci. Pract..

[189] Tan Z.Y., Bealgey K.W., Fang Y., Gong Y.M., Bao S. (2009). Interleukin-23: Immunological roles and clinical implications. Int. J. Biochem. Cell Biol..

[190] Lee Y., Awasthi A., Yosef N., Quintana F.J., Xiao S., Peters A., Wu C., Kleinewietfeld M., Kunder S., Hafler D.A. (2012). Induction and molecular signature of pathogenic TH17 cells. Nat. Immunol..

[191] Tang C., Chen S., Qian H., Huang W. (2012). Interleukin-23: As a drug target for autoimmune inflammatory diseases. Immunology.

[192] Schmidt C., Giese T., Ludwig B., Mueller-Molaian I., Marth T., Zeuzem S., Meuer S.C., Stallmach A. (2005). Expression of interleukin-12-related cytokine transcripts in inflammatory bowel disease: Elevated interleukin-23p19 and interleukin-27p28 in Crohn’s disease but not in ulcerative colitis. Inflamm. Bowel Dis..

[193] Cici D., Corrado A., Rotondo C., Cantatore F.P. (2019). Wnt Signaling and biological therapy in rheumatoid arthritis and spondyloarthritis. Int. J. Mol. Sci..

[194] Saito-Diaz K., Chen T.W., Wang X., Thorne C.A., Wallace H.A., Page-McCaw A., Lee E. (2013). The way Wnt works: Components and mechanism. Growth Factors.

[195] Maruotti N., Corrado A., Neve A., Cantatore F.P. (2013). Systemic effects of Wnt signaling. J. Cell. Physiol..

[196] Uluçkan Ö., Jimenez M., Karbach S., Jeschke A., Graña O., Keller J., Busse B., Croxford A.L., Finzel S., Koenders M. (2016). Chronic skin inflammation leads to bone loss by IL-17–mediated inhibition of Wnt signaling in osteoblasts. Sci. Transl. Med..

[197] Heiland G.R., Zwerina K., Baum W., Kireva T., Distler J.H., Grisanti M., Asuncion F., Li X., Ominsky M., Richards W. (2010). Neutralisation of Dkk-1 protects from systemic bone loss during inflammation and reduces sclerostin expression. Ann. Rheum. Dis..

[198] Lim S.Y., Bolster M. (2017). Profile of romosozumab and its potential in the management of osteoporosis. Drug Des. Devel. Ther..

[199] McClung M.R., Grauer A., Boonen S., Bolognese M.A., Brown J.P., Diez-Perez A., Langdahl B.L., Reginster J.-Y., Zanchetta J.R., Wasserman S.M. (2014). Romosozumab in postmenopausal women with low bone mineral density. N. Engl. J. Med..

[200] Cosman F., Crittenden D.B., Ferrari S., Khan A., Lane N.E., Lippuner K., Matsumoto T., Milmont C.E., Libanati C., Grauer A. (2018). FRAME Study: The foundation effect of building bone with 1 year of romosozumab leads to continued lower fracture risk after transition to denosumab. J. Bone Miner. Res..

[201] McClung M.R., Brown J.P., Diez-Perez A., Resch H., Caminis J., Meisner P., Bolognese M.A., Goemaere S., Bone H.G., Zanchetta J.R. (2018). Effects of 24 months of treatment with romosozumab followed by 12 months of denosumab or placebo in postmenopausal women with low bone mineral density: A randomized, double-blind, phase 2, parallel group study. J. Bone Miner. Res..

[202] Grebennikova T.A., Belaya Z.E., Rozhinskaya L.Y., Melnichenko G.A. (2016). The canonical Wnt/β-catenin pathway: From the history of its discovery to clinical application. Ter. Arkh..

[203] Pietrzyk B., Smertka M., Chudek J. (2017). Sclerostin: Intracellular mechanisms of action and its role in the pathogenesis of skeletal and vascular disorders. Adv. Clin. Exp. Med..

[204] Poole K.E.S., Van Bezooijen R.L., Loveridge N., Hamersma H., Papapoulos S.E., Löwik C.W., Reeve J. (2005). Sclerostin is a delayed secreted product of osteocytes that inhibits bone formation. FASEB J..

[205] Xiong J., Piemontese M., Onal M., Campbell J., Goellner J.J., Dusevich V., Bonewald L., Manolagas S.C., O’Brien C.A. (2015). Osteocytes, not osteoblasts or lining cells, are the main source of the RANKL required for osteoclast formation in remodeling bone. PLoS ONE.

[206] Singh A., Gupta M.K., Mishra S.P. (2019). Study of correlation of level of expression of Wnt signaling pathway inhibitors sclerostin and dickkopf-1 with disease activity and severity in rheumatoid arthritis patients. Drug Discov. Ther..

[207] Brandenburg V.M., D’Haese P., Deck A., Mekahli D., Meijers B., Neven E., Evenepoel P. (2016). From skeletal to cardiovascular disease in 12 steps—The evolution of sclerostin as a major player in CKD-MBD. Pediatr. Nephrol..

[208] Diarra D., Stolina M., Polzer K., Zwerina J., Ominsky M.S., Dwyer D., Korb A., Smolen J., Hoffmann M., Scheinecker C. (2007). Dickkopf-1 is a master regulator of joint remodeling. Nat. Med..

[209] Daoussis D., Liossis S.N.C., Solomou E.E., Tsanaktsi A., Bounia K., Karampetsou M., Yiannopoulos G., Andonopoulos A.P. (2010). Evidence that Dkk-1 is dysfunctional in ankylosing spondylitis. Arthritis Rheum..

[210] Klingberg E., Nurkkala M., Carlsten H., Forsblad-D’Elia H. (2014). Biomarkers of bone metabolism in ankylosing spondylitis in relation to osteoproliferation and osteoporosis. J. Rheumatol..

[211] Gatti D., Viapiana O., Idolazzi L., Fracassi E., Ionescu C., Dartizio C., Troplini S., Kunnathully V., Adami S., Rossini M. (2014). Distinct effect of zoledronate and clodronate on circulating levels of DKK1 and sclerostin in women with postmenopausal osteoporosis. Bone.

[212] Gatti D., Viapiana O., Fracassi E., Idolazzi L., Dartizio C., Povino M.R., Adami S., Rossini M. (2012). Sclerostin and DKK1 in postmenopausal osteoporosis treated with denosumab. J. Bone Miner. Res..

[213] Tai N., Inoue D. (2014). Anti-Dickkopf1 (Dkk1) antibody as a bone anabolic agent for the treatment of osteoporosis. Clin. Calcium.

[214] Szentpétery Á., Horváth Á., Gulyás K., Pethö Z., Bhattoa H.P., Szántó S., Szücs G., FitzGerald O., Schett G., Szekanecz Z. (2017). Effects of targeted therapies on the bone in arthritides. Autoimmun. Rev..

[215] Ramazani Y., Knops N., Elmonem M.A., Nguyen T.Q., Arcolino F.O., van den Heuvel L., Levtchenko E., Kuypers D., Goldschmeding R. (2018). Connective tissue growth factor (CTGF) from basics to clinics. Matrix Biol..

[216] Rosenbloom J., Macarak E., Piera-Velazquez S., Jimenez S.A., Fibrosis R.L. (2017). Human fibrotic diseases: Current challenges in fibrosis research. Methods in Molecular Biology.

[217] Varga J., Trojanowska M., Kuwana M. (2017). Pathogenesis of systemic sclerosis: Recent insights of molecular and cellular mechanisms and therapeutic opportunities. J. Scleroderma Relat. Disord..

[218] Makino K., Makino T., Stawski L., Lipson K.E., Leask A., Trojanowska M. (2017). Anti-connective tissue growth factor (CTGF/CCN2) monoclonal antibody attenuates skin fibrosis in mice models of systemic sclerosis. Arthritis Res. Ther..

[219] Parapuram S.K., Shi-wen X., Elliott C., Welch I.D., Jones H., Baron M., Denton C.P., Abraham D.J., Leask A. (2011). Loss of PTEN expression by dermal fibroblasts causes skin fibrosis. J. Invest. Dermatol..

[220] Agnholt J., Kelsen J., Schack L., Hvas C.L., Dahlerup J.F., Sørensen E.S. (2007). Osteopontin, a protein with cytokine-like properties, is associated with inflammation in Crohn’s disease. Scand. J. Immunol..

[221] Sodek J., Ganss B., McKee M.D. (2000). Osteopontin. Crit. Rev. Oral Biol. Med..

[222] Ishijima M., Rittling S.R., Yamashita T., Tsuji K., Kurosawa H., Nifuji A., Denhardt D.T., Noda M. (2001). Enhancement of osteoclastic bone resorption and suppression of osteoblastic bone formation in response to reduced mechanical stress do not occur in the absence of osteopontin. J. Exp. Med..

[223] Thurner P.J., Chen C.G., Ionova-Martin S., Sun L., Harman A., Porter A., Ager J.W., Ritchie R.O., Alliston T. (2010). Osteopontin deficiency increases bone fragility but preserves bone mass. Bone.

[224] Fan Y., He R., Zou L., Meng J. (2020). [Clinical value of biomarkers in diagnosis and treatment of idiopathic pulmonary fibrosis]. Nan Fang Yi Ke Da Xue Xue Bao.

[225] Wu P.-M., Lin C.-H., Lee H.-T., Shih H.-I., Huang C.-C., Tu Y.-F. (2020). Early blood biomarkers distinguish inflammation from neonatal hypoxic-ischemia encephalopathy. Neurochem. Res..

[226] Yazici O., Dogan M., Ozal G., Aktas S.H., Demirkazik A., Utkan G., Senler F.C., Icli F., Akbulut H. (2020). Osteopontin is a prognostic factor in patients with advanced gastric cancer. Comb. Chem. High. Throughput Screen..

[227] Smith E.A., Krumpelbeck E.F., Jegga A.G., Prell M., Matrka M.M., Kappes F., Greis K.D., Ali A.M., Meetei A.R., Wells S.I. (2018). The nuclear DEK interactome supports multi-functionality. Proteins Struct. Funct. Bioinforma..

[228] Dong X., Wang J., Kabir F.N., Shaw M., Reed A.M., Stein L., Andrade L.E.C., Trevisani V.F.M., Miller M.L., Fujii T. (2000). Autoantibodies to DEK oncoprotein in human inflammatory disease. Arthritis Rheum..

[229] Audrito V., Messana V.G., Deaglio S. (2020). NAMPT and NAPRT: Two metabolic enzymes with key roles in inflammation. Front. Oncol..

[230] Franco-Trepat E., Alonso-Pérez A., Guillán-Fresco M., Jorge-Mora A., Gualillo O., Gómez-Reino J.J., Gómez Bahamonde R. (2019). Visfatin as a therapeutic target for rheumatoid arthritis. Expert Opin. Ther. Targets.

[231] Vandooren J., Van den Steen P.E., Opdenakker G. (2013). Biochemistry and molecular biology of gelatinase B or matrix metalloproteinase-9 (MMP-9): The next decade. Crit. Rev. Biochem. Mol. Biol..

[232] Ram M., Sherer Y., Shoenfeld Y. (2006). Matrix metalloproteinase-9 and autoimmune diseases. J. Clin. Immunol..

[233] Blair J.P.M., Bager C., Platt A., Karsdal M., Bay-Jensen A.-C. (2019). Identification of pathological RA endotypes using blood-based biomarkers reflecting tissue metabolism. A retrospective and explorative analysis of two phase III RA studies. PLoS ONE.

[234] Park S.G., Jeong S.U., Lee J.H., Ryu S.H., Jeong H.J., Sim Y.J., Kim D.K., Kim G.C. (2018). The changes of ctx, dpd, osteocalcin, and bone mineral density during the postmenopausal period. Ann. Rehabil. Med..

[235] Banshchikova N.Y., Letyagina Y.A., Omelchenko V.O., Korolev M.A. (2018). Antiresorptive activity of denosumab in the treatment of osteoporosis in patients with rheumatoid arthritis. Osteoporos. Bone Dis..

[236] Bay-Jensen A.C., Platt A., Siebuhr A.S., Christiansen C., Byrjalsen I., Karsdal M.A. (2016). Early changes in blood-based joint tissue destruction biomarkers are predictive of response to tocilizumab in the LITHE study. Arthritis Res. Ther..

[237] Segal A.W. (2005). How neutrophils kill microbes. Annu. Rev. Immunol..

[238] Korkmaz B., Hajjar E., Kalupov T., Reuter N., Brillard-Bourdet M., Moreau T., Juliano L., Gauthier F. (2007). Influence of Charge Distribution at the Active Site Surface on the Substrate Specificity of Human Neutrophil Protease 3 and Elastase. J. Biol. Chem..

[239] Reumaux D., Duthilleul P., Roos D. (2004). Pathogenesis of diseases associated with antineutrophil cytoplasm autoantibodies. Hum. Immunol..

[240] Kallenberg C.G.M. (2008). Pathogenesis of PR3-ANCA associated vasculitis. J. Autoimmun..

[241] Kallenberg C.G., Heeringa P., Stegeman C.A. (2006). Mechanisms of Disease: Pathogenesis and treatment of ANCA-associated vasculitides. Nat. Clin. Pract. Rheumatol..

[242] Kessenbrock K., Krumbholz M., Schönermarck U., Back W., Gross W.L., Werb Z., Gröne H.-J., Brinkmann V., Jenne D.E. (2009). Netting neutrophils in autoimmune small-vessel vasculitis. Nat. Med..

[243] Petrini I. (2015). Biology of MET: A double life between normal tissue repair and tumor progression. Ann. Transl. Med..

[244] Grano M., Galimi F., Zambonin G., Colucci S., Cottone E., Zallone A.Z., Comoglio P.M. (1996). Hepatocyte growth factor is a coupling factor for osteoclasts and osteoblasts in vitro. Proc. Natl. Acad. Sci. USA.

[245] Nagashima M., Hasegawa J., Kato K., Yamazaki J., Nishigai K., Ishiwata T., Asano G., Yoshino S. (2001). Hepatocyte growth factor (HGF), HGF activator, and c-Met in synovial tissues in rheumatoid arthritis and osteoarthritis. J. Rheumatol..

[246] Sugiura T., Kawaguchi Y., Soejima M., Katsumata Y., Gono T., Baba S., Kawamoto M., Murakawa Y., Yamanaka H., Hara M. (2010). Increased HGF and c-Met in muscle tissues of polymyositis and dermatomyositis patients: Beneficial roles of HGF in muscle regeneration. Clin. Immunol..

[247] Torres L., Klingberg E., Nurkkala M., Carlsten H., Forsblad-d’Elia H. (2019). Hepatocyte growth factor is a potential biomarker for osteoproliferation and osteoporosis in ankylosing spondylitis. Osteoporos. Int..

[248] Navarini L., Margiotta D.P.E., Vadacca M., Afeltra A. (2018). Leptin in autoimmune mechanisms of systemic rheumatic diseases. Cancer Lett..

[249] Diaz-Rizo V., Bonilla-Lara D., Gonzalez-Lopez L., Sanchez-Mosco D., Fajardo-Robledo N.S., Perez-Guerrero E.E., Rodriguez-Jimenez N.A., Saldaña-Cruz A.M., Vazquez-Villegas M.L., Gomez-Bañuelos E. (2017). Serum levels of adiponectin and leptin as biomarkers of proteinuria in lupus nephritis. PLoS ONE.

[250] Batún-Garrido J.A. (2018). de J.; Salas-Magaña, M.; Juárez-Rojop, I.E.; Hernández-Núñez, E.; Olán, F. Relación entre las concentraciones de la leptina y la actividad de la enfermedad en pacientes con artritis reumatoide. Med. Clin..

[251] Abella V., Scotece M., Conde J., Pino J., Gonzalez-Gay M.A., Gómez-Reino J.J., Mera A., Lago F., Gómez R., Gualillo O. (2017). Leptin in the interplay of inflammation, metabolism and immune system disorders. Nat. Rev. Rheumatol..

[252] Schäffler A., Ehling A., Neumann E., Herfarth H., Tarner I., Schölmerich J., Müller-Ladner U., Gay S. (2003). Adipocytokines in synovial fluid. JAMA.

[253] Sglunda O., Mann H., Hulejová H., Kuklová M., Pecha O., Pleštilová L., Filková M., Pavelka K., Vencovský J., Šenolt L. (2014). Decreased circulating visfatin is associated with improved disease activity in early rheumatoid arthritis: Data from the PERAC cohort. PLoS ONE.

[254] Batún-Garrido J.A.D.J., Salas-Magaña M., Juárez-Rojop I.E. (2018). Association between leptin and IL-6 concentrations with cardiovascular risk in patients with rheumatoid arthritis. Clin. Rheumatol..

[255] Stawski L., Trojanowska M. (2019). Oncostatin M and its role in fibrosis. Connect. Tissue Res..

[256] Rajashekhar G., Willuweit A., Patterson C.E., Sun P., Hilbig A., Breier G., Helisch A., Clauss M. (2006). Continuous endothelial cell activation increases angiogenesis: Evidence for the direct role of endothelium linking angiogenesis and inflammation. J. Vasc. Res..

[257] Hermanns H.M. (2015). Oncostatin M and interleukin-31: Cytokines, receptors, signal transduction and physiology. Cytokine Growth Factor Rev..

[258] West N.R., Hegazy A.N., Owens B.M.J., Bullers S.J., Linggi B., Buonocore S., Coccia M., Görtz D., This S., Stockenhuber K. (2017). Oncostatin M drives intestinal inflammation and predicts response to tumor necrosis factor–neutralizing therapy in patients with inflammatory bowel disease. Nat. Med..

[259] McGarry T., Orr C., Wade S., Biniecka M., Wade S., Gallagher L., Low C., Veale D.J., Fearon U. (2018). JAK/STAT Blockade alters synovial bioenergetics, mitochondrial function, and proinflammatory mediators in rheumatoid arthritis. Arthritis Rheumatol..

[260] Fearon U., Mullan R., Markham T., Connolly M., Sullivan S., Poole A.R., FitzGerald O., Bresnihan B., Veale D.J. (2006). Oncostatin M induces angiogenesis and cartilage degradation in rheumatoid arthritis synovial tissue and human cartilage cocultures. Arthritis Rheum..

[261] Langdon C., Kerr C., Hassen M., Hara T., Arsenault A.L., Richards C.D. (2000). Murine Oncostatin M stimulates mouse synovial fibroblasts in vitro and induces inflammation and destruction in mouse joints in vivo. Am. J. Pathol..

[262] Hanlon M.M., Rakovich T., Cunningham C.C., Ansboro S., Veale D.J., Fearon U., McGarry T. (2019). STAT3 mediates the differential effects of oncostatin M and TNFα on RA synovial fibroblast and endothelial cell function. Front. Immunol..

[263] Ochsner U.A., Green L.S., Gold L., Janjic N. (2014). Systematic selection of modified aptamer pairs for diagnostic sandwich assays. Biotechniques.

[264] Chen K., Zhou J., Shao Z., Liu J., Song J., Wang R., Li J., Tan W. (2020). Aptamers as versatile molecular tools for antibody production monitoring and quality control. J. Am. Chem. Soc..

[265] Wildner S., Huber S., Regl C., Huber C.G., Lohrig U., Gadermaier G. (2019). Aptamers as quality control tool for production, storage and biosimilarity of the anti-CD20 biopharmaceutical rituximab. Sci. Rep..

[266] Kohlberger M., Wildner S., Regl C., Huber C.G., Gadermaier G. (2020). Rituximab-specific DNA aptamers are able to selectively recognize heat-treated antibodies. PLoS ONE.

